# Triplet Photosensitized *para*-Hydrogen Induced Polarization

**DOI:** 10.1021/acscentsci.2c01003

**Published:** 2022-11-14

**Authors:** Emily E. Brown, Iuliia Mandzhieva, Patrick M. TomHon, Thomas Theis, Felix N. Castellano

**Affiliations:** Department of Chemistry, North Carolina State University, Raleigh, North Carolina 27965-8204, United States

## Abstract

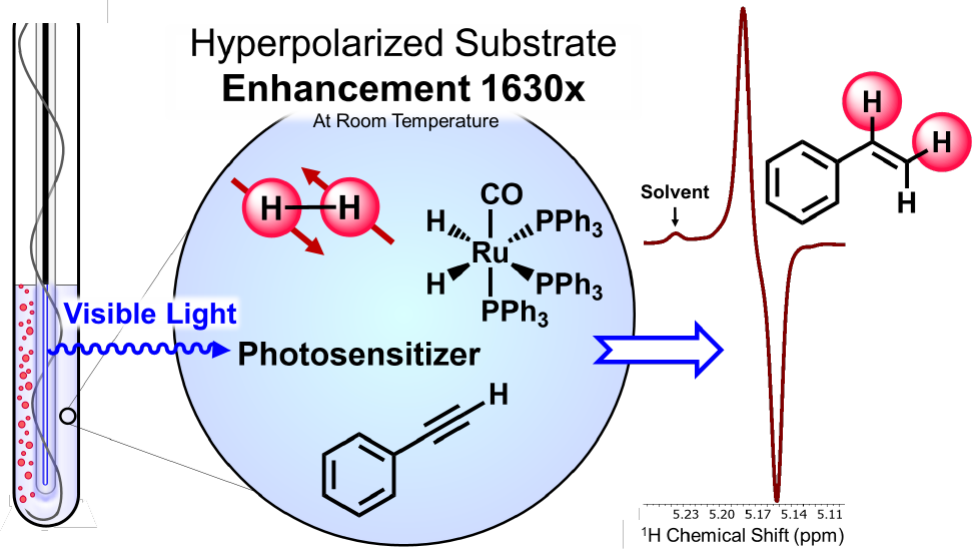

Despite its enormous utility in structural characterization, nuclear magnetic resonance (NMR) spectroscopy is inherently limited by low spin polarization. One method to address the low polarization is *para*-hydrogen (*p*-H_2_) induced polarization (PHIP) which uses the singlet spin isomer of H_2_ to generate disparate nuclear spin populations to amplify the associated NMR signals. PHIP often relies on thermal catalysis or, more infrequently, UV-activated catalytic hydrogenation. Light-activated hydrogenation enables direct and timed control over the hyperpolarization of target substrates, critical for identifying short-lived intermediates. Here, we use an established Ir(III) triplet photosensitizer (**PS**) to visible light sensitize the triplet ligand-field states in the d^6^-transition metal dihydride Ru(CO)(PPh_3_)_3_(H)_2_ (**1**). Excitation inside a 9.4 T NMR spectrometer with the **PS** and a 420 nm blue LED, under 3 atm of *p*-H_2_, successfully photosensitized hyperpolarization in **1** and in a range of unsaturated substrates at and below room temperature, up to 1630-fold. In otherwise identical experimental conditions without light activation, no polarization was realized in **1** or the substrates evaluated. We believe triplet-sensitized PHIP (Trip-PHIP) represents a facile experimental means for probing triplet sensitized light activation in transition metal catalysts possessing low-lying triplet ligand-field states, providing mechanistic insight of potentially tremendous value in chemical catalysis.

## Abstract 2

This work demonstrates the use of an Ir(III) triplet photosensitizer enabling visible light activated hyperpolarization from *para*-hydrogen—yielding NMR signal enhancements up to 1630-fold in a Ru(II) homogeneous dihydride catalyst as well as a range of hydrogenation substrates at and below room temperature.

## Introduction

Nuclear magnetic resonance (NMR) spectroscopy is a ubiquitous method for determining molecular structure across myriad scientific fields. Despite the widespread utility of NMR, it is greatly undermined by low spin polarization due to the near equivalence of nuclear spin populations at and near room temperature. Achieving acceptable signal detection and frequency resolution requires high magnetic fields, long experimental run times, and comparatively concentrated samples.^1^ Quality NMR spectra can take hours to days to measure, and accessible methods of increasing sample polarization, such as decreasing the temperature and increasing the magnetic field, provide only meager polarization enhancement. More rapid hyperpolarization approaches exist, including *para*-hydrogen based methods such as *para*-hydrogen (*p*-H_2_) induced polarization (PHIP).^2^ First unequivocally demonstrated in 1987 by Bowers and Weitekamp and several months later by Eisenburg, Balch et al.,^2,3^^1^H NMR hyperpolarization was generated using the singlet nuclear spin isomer *p*-H_2_ and a homogeneous thermally activated transition metal containing hydrogenation catalyst. PHIP is a relatively accessible method of increasing nuclear polarization, and often depends on thermal oxidative addition of *p*-H_2_ to a transition metal complex, frequently in the form of a transition metal hydride that subsequently transfers this polarization to an organic hydrogenation substrate.^4−7^ Hyperpolarization from *p*-H_2_ can generate more than 13% polarization^5^ corresponding to over 37,000-fold enhancement with respect to thermal polarization at 1 T (43 MHz). UV activated photocatalytic PHIP has been recently demonstrated at room temperature and above, specifically hydrogenation of diphenylacetylene using ruthenium(II) arsine carbonyl dihydrides.^8^ Great advances have been made in hyperpolarizing substrates utilizing *p*-H_2_ in concert with thermal hydrogenation catalysts that circumvent the use of extended signal averaging, concentrated samples, and high magnetic fields.^1,9^

*para*-Hydrogen based methods leveraging thermal exchange or hydrogenation lack the “turn-on” activation control imparted by light-driven methods. Unlike approaches utilizing thermal energy, motifs featuring light activation can selectively transfer energy to a desired species, thereby activating the intended reaction. Photochemistry has the advantage of more than 50 years of knowledge regarding the photoelimination of hydrides and their reformation from transition metal catalysts in room temperature solutions.^10−12^ Previous research has incorporated the photoactivated elimination of metal dihydrides into the realm of PHIP, utilizing Ru(II) transition metal dihydrides and deep UV light excitation at and above room temperature.^8,13−15^ A prominent example involves the direct excitation of Ru(CO)(PPh_3_)_3_(H)_2_ (**1**) in the presence of *p*-H_2_ utilizing the well-known photochemical loss of H_2_ and the subsequent reformation of the dihydride in excess *p*-H_2_, resulting in hyperpolarization of the regenerated metal hydride.^15,16^ Numerous studies have demonstrated photoactivated H_2_ loss from **1**, but to date this H_2_ elimination approach has been constrained to UV light excitation.^13,14,16,17^

In addition to its H_2_-generating photoactivity, **1** is a prolific cross-coupling catalyst, first demonstrated in the Murai reaction,^18,19^ where an ortho carbon–carbon bond is formed between an aromatic species with an oxygen directing group and an olefin or acetylene.^19−21^ Primary catalytic applications of **1** include cross-coupling reactions between species ranging from aromatic and nonaromatic ketones and olefins,^22−27^ to aromatic ketones and arylborates,^28−31^ as well as catalyzing heterocycle formation,^32−35^ dimerization,^36,37^ additions on the 2,5,8,11 position of perylene bisimide,^31,38^ H_2_ production,^39^ and as a reactant for other catalytic species.^40−43^ This catalyst has also been demonstrated to execute H/D exchange.^24,44^ Interestingly, the use of **1** as a hydrogenation catalyst has been underdeveloped, where hydrogenation was originally demonstrated as an intermediate reaction step,^24,27,41^ and **1**, among other ruthenium-based catalysts, was screened for hydrogenation activity.^45^ Molecule **1** was utilized for the hydrogenation of 1,5,9-cyclododecatriene in toluene at 140 °C with mediocre selectivity and total catalyst decomposition over 5 h.^45^ While the thermal catalytic activity for **1** has been extensively documented, no photocatalysis, least of all hydrogenation, has ever been reported to the best of our knowledge.

**Scheme 1:**
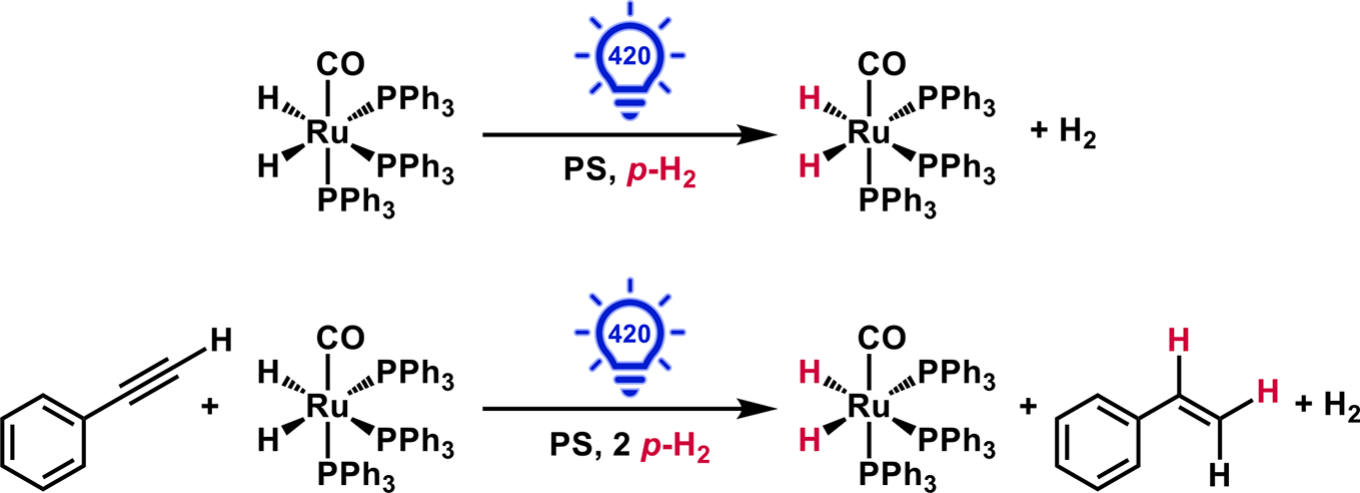
Photoactivated Hydride Exchange in Ru(CO)(PPh_3_)_3_(H)_2_ (Top) and Photocatalytic Hydrogenation of Phenylacetylene by **1 (**Bottom) Derived from the Trip-PHIP Process in Conjunction with a **PS** Using 420 nm Excitation at and below Room Temperature in Dichloromethane

All low-spin d^6^-based octahedral transition metal complexes possess low lying triplet ligand-field states that are antibonding with respect to metal–ligand bonds. It is these states that are most likely populated in both thermal and photochemical reactions of homogeneous transition metal dihydrides. Therefore, we postulated that photosensitization of the triplet ligand-field states in a Ru(II) dihydride could also be accomplished using only visible light with an energetically appropriate triplet sensitizer. Subsequently, if H_2_ loss could be triplet sensitized, visible light-induced photoactivated hydrogenation catalysts would likely result, opening a new area of scientific research. Here, we demonstrate triplet photosensitization of **1** using [Ir(dF(CF_3_)ppy)_2_(dtbbpy)]PF_6_ (**PS**), where dF(CF_3_)ppy is 2-(2,4-difluorophenyl)-5-(trifluoromethyl)pyridine, and dtbbpy is 4,4′-di-*tert*-butyl-2,2′-bipyridine, in conjunction with 420 or 427 nm blue light excitation in the absence and presence of a number of hydrogenation substrates, compared with samples energized using UVA light (365 nm) in the absence of the **PS**, Scheme 1. This strategy successfully photosensitized ^1^H NMR hyperpolarization in **1** in addition to a range of hydrogenation substrates at and below room temperature enabling facile detection of the resulting reaction products.

## Results and Discussion

### Triplet Photosensitization H_2_ Elimination in **1**

Traditional hydrogenative PHIP primarily depends on thermal hydrogenation or hydride exchange which offers diminished control over the addition of *para*-hydrogen to a target substrate.^4−7^ Using visible light to sensitize the reaction can enable the generation of hyperpolarization with greater selectivity and efficiency since other intervening excited states are bypassed. Also, photocontrolled PHIP enables much tighter experimental control in the time domain to elicit coherent spin dynamics instead of incoherent averaging over randomly distributed hydrogenation events in time.^13,46^ The efficacy of the triplet photosensitization depends on an energetically matched pair of photosensitizer and quencher, here **PS** and **1**. Compound **1** is not photoluminescent at room temperature or at 77 K, which necessitated an alternative approach to estimate the triplet ligand field state energies. Gas phase DFT calculations (Gaussian 16) were used to approximate the triplet ligand field energy of **1** near 1.9 eV, which was estimated by optimizing the geometry and energies of the singlet ground state and the first excited triplet state, Figure S1. The previously published optimized geometry of **1** was used as the starting point for these calculations.^14^ All low-lying triplet ligand-field states in **1** are expected to be dissociative, populating metal–ligand σ* orbitals, consistent with the known photoelimination of H_2_ under UV-light exposure.^15^

**Figure 1:**
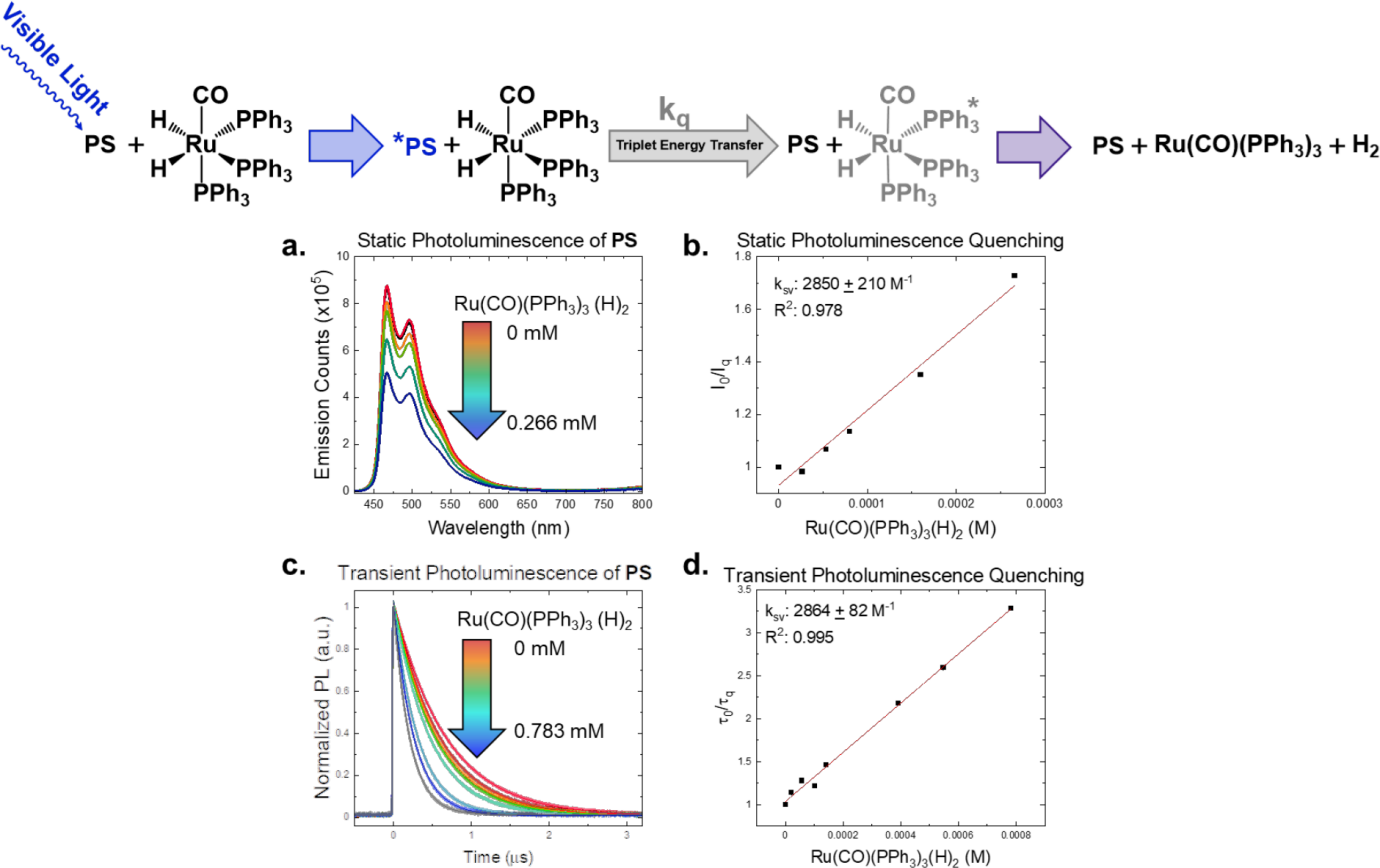
Dynamic Stern–Volmer quenching of the **PS** by **1**, using static (a, b) and transient photoluminescence (c, d), all excited at 420 nm in CH_2_Cl_2,_ along with the proposed triplet photosensitized reductive elimination of H_2_ from **1** presented at the top.

The high energy Ir(III) **PS** (Scheme 1, *E*_T_ = 2.6 eV)^47^ was paired with dihydride **1** to ensure sufficient driving force for triplet–triplet energy transfer (TTET) to selectively populate the requisite triplet ligand field states.^48^ The rate constant of the near diffusion-limited TTET quenching (1.39 ± 0.21 × 10^9^ M^–1^ s^–1^) in aerated CH_2_Cl_2_ was measured using both static and dynamic photoluminescence experiments, Figure 1, yielding linear Stern–Volmer plots in both instances. These data are consistent with dynamic quenching of the **PS** excited state by **1**.^49^ Once excited with 355 or 305 nm, the resultant Ru(0) intermediate has a characteristic absorption maximum at 380 nm.^16^ Nanosecond transient absorption experiments demonstrate that blue nanosecond laser pulse excitation of the **PS** in dichloromethane indeed sensitizes the loss of both hydride ligands in **1** (Figure S2), also producing the characteristic Ru(0) intermediate. To further ensure that visible light photosensitization leads to loss of H_2_ from **1**, a solution containing both the **PS** and **1** in an NMR tube was irradiated at 427 nm for 5 min using a blue Kessil lamp in aerated CD_2_Cl_2_. Clear loss of the hydride resonances was detected in the resultant ^1^H NMR spectrum, Figure S3.

### Triplet Sensitization of PHIP in Ru(CO)(PPh_3_)_3_(H)_2_ (**1**)

**Figure 2:**
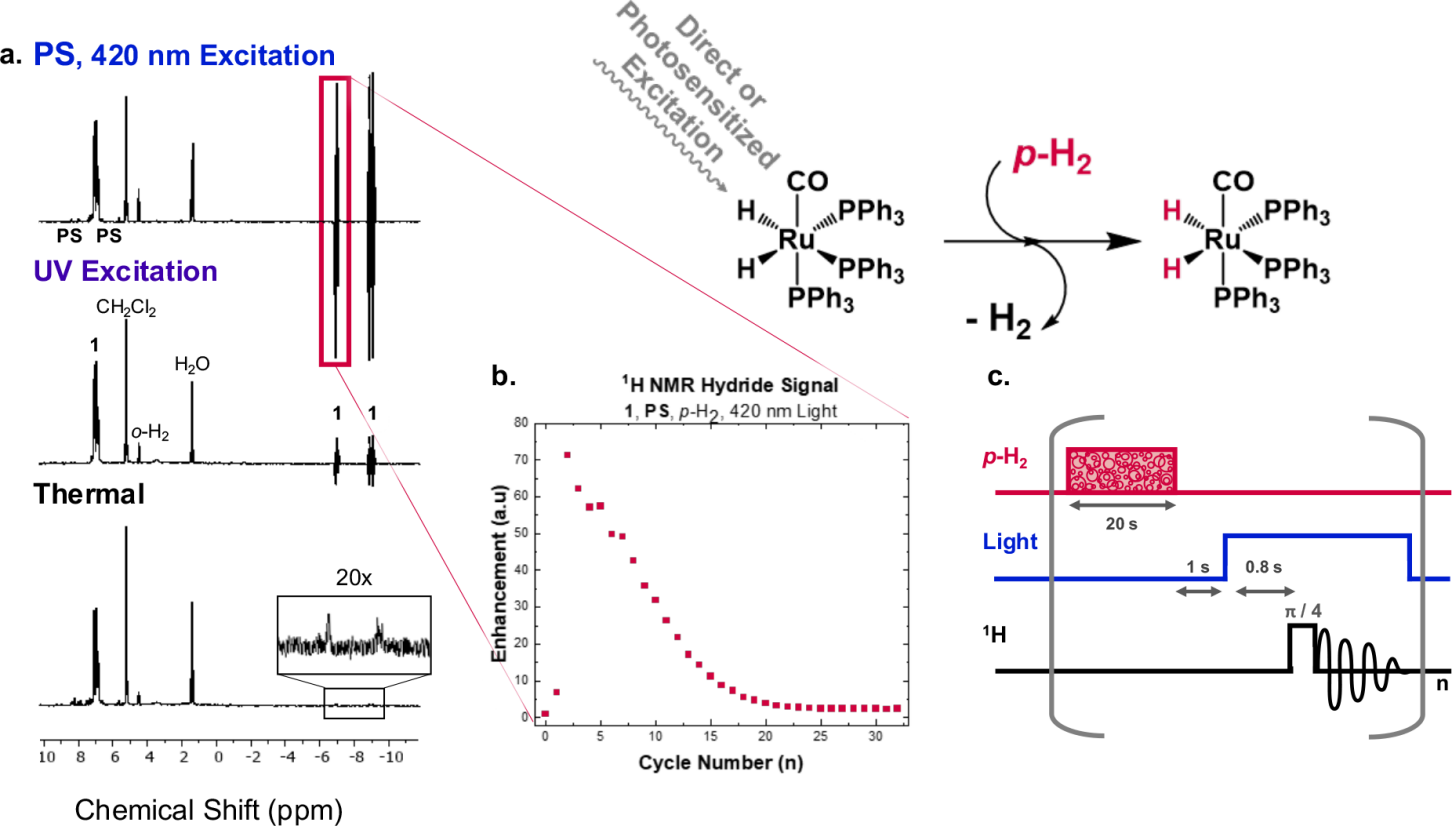
(a) ^1^H NMR spectra of **1** (single scans) measured in CD_2_Cl_2_ at 400 MHz under a variety of experimental conditions in the presence of 3 atm *p*-H_2_ using the pulse acquisition sequence depicted in (c): **1** and **PS** under 420 nm blue LED excitation that leads to the greatest signal enhancement (top); **1** in the presence of 365 nm UV LED excitation (center), and a thermally equilibrated sample of **1** and **PS** measured in the absence of light activation (bottom). (b) Integrated ^1^H NMR signal in the metal-hydride region (−6 ppm) measured as a function of each single acquisition cycle from the triplet photosensitized reaction mixture with 420 nm LED excitation. (c) Depiction of the pulse program used that automated the timing of the bubbling of *p*-H_2_ in the NMR tube, light excitation, and the application of the 45° RF pulse. Please see the Supporting Information for specific experimental details.

The photochemically reversible loss of the two hydride ligands from **1**’s excited state has been leveraged previously to achieve hyperpolarization in **1** by reforming the dihydride species in the presence of *p*-H_2_.^13,14,17^ While previous work focused on sophisticated experiments using pulsed UV laser irradiation *in situ*, more recent developments in fiber optic-based approaches enable widespread application of photoactivated processes detected by NMR spectroscopy.^50−52^ A fiber optic cable whose cladding was sanded to enable irradiation of the NMR sample across the entire probe region (Figure S4) facilitated the *in situ* measurements described here. Direct excitation of **1** at 365 nm yielded up to a total 35.6-fold enhancement over all the cycles of a run (Figure 2a), in the ^1^H NMR hydride resonances in the presence of 3 atm of *p*-H_2_, consistent with observations from 305 or 355 nm light excitation.^13,14,16,17^ For the hydrides, enhancement was calculated by combining the hyperpolarized signal of multiple cycles of a run and comparing it to the thermal signal taken before the cycles of light and *p*-H_2_, measured under the same conditions; please see the Supporting Information for details. Using the Ir(III) photosensitizer and 420 nm light in the presence of **1**, markedly increased hyperpolarization (235-fold over thermal polarization) was realized under identical experimental conditions (Figure 2a), illustrating that triplet-sensitized PHIP (Trip-PHIP) selectively targets the antibonding triplet ligand field states. Regardless of the excitation wavelength, the resulting hyperpolarized signals decay, but they can be rapidly regenerated in repetitive cycling experiments as shown in Figure 2b. Figure 2c depicts the cyclical pulse sequence (bubbling *p*-H_2_, irradiating the sample, application of the RF pulse and data acquisition) applied here with each data point in Figure 2b corresponding to a single scan. The decreasing signal intensity observed as a function of time is influenced by established deleterious side photoreactions and likely includes the loss of a triphenylphosphine ligand, dimerization, η^2^-coordination of dihydrogen, and other unidentified reaction pathways.^14^

### Reaction of Photoactivated **1** with Phenylacetylene

**Figure 3:**
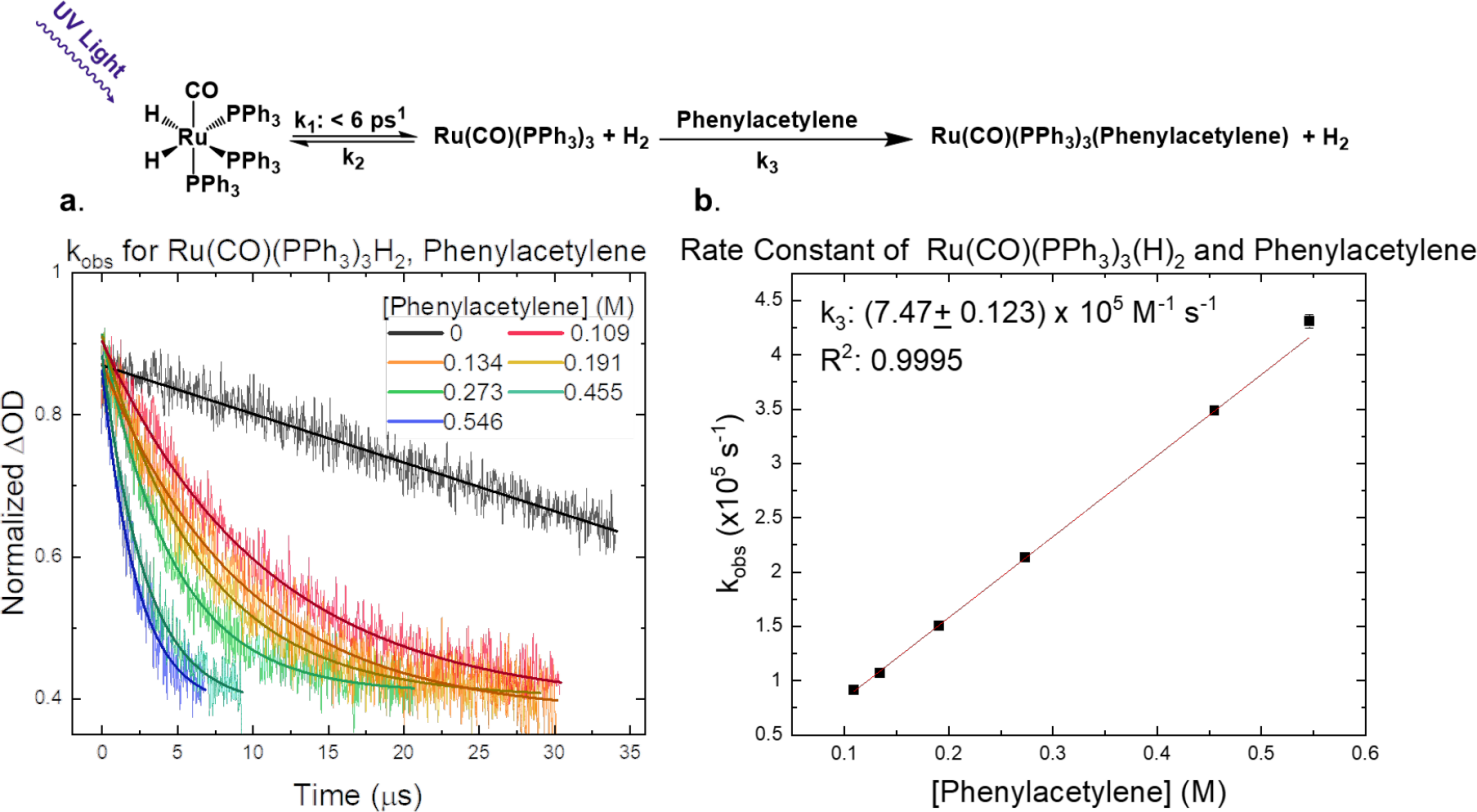
(Top) The proposed sequence of reaction steps following light excitation of **1** in the presence of phenylacetylene; the rate constant *k*_1_ was taken from previous work.^16^ (a) Time-resolved absorption decay kinetics of the Ru(0) intermediate at 380 nm measured as a function of phenylacetylene concentration following 355 nm pulsed laser excitation (7 ns fwhm) measured in aerated dichloromethane with the corresponding single exponential fits displayed as a solid line superimposed on each transient decay. (b) The calculated rate constant for the reaction occurring between the Ru(0) intermediate and phenylacetylene, using the data from (a) obtained under pseudo-first-order conditions.

While the interactions of hydride **1** with light are well established, the applications of **1** in the realm of photocatalysis are unexplored.^13−15^ While the Ru(II) dihydride used in this work (**1**) is known to engage in hydrogenation reactions at high temperatures, catalysis research has primarily focused on **1** facilitating cross-coupling reactions.^18,19,23−29,31,45,53^ The reductive elimination and photorelease of H_2_ from **1** upon direct excitation, *k*_1_ < 6 ps, are established, as well as the photosensitized release of H_2_, Figure S3.^16^ As the subsequent reformation of the **1** in the presence of H_2_ is also well documented, (*k*_2_ = 8.4 ± 0.4 × 10^7^ M^–1^ s^–1^), we decided to explore whether **1** could photocatalytically hydrogenate substrates at and below room temperature.^13,14,16,17^ To first glean insight into any potential reaction, we monitored the interaction of phenylacetylene with the photogenerated Ru(0) intermediate. Previous literature bolsters this approach as similar ruthenium dihydrides featuring bidentate chelating phosphines were likewise monitored upon light excitation demonstrating quenching of the respective Ru(0) intermediates in the presence of various substrates and ligands.^54,55^ In those molecules, the rate constant for oxidative addition of H_2_ was rather fast, on the order of 10^10^, 10^8^, and 10^7^ M^–1^ s^–1^ for the methyl-, ethyl-, and phenyl-substituted chelating phosphines, respectively. However, in the presence of ethylene, the second-order rate constant for the reformation of the original Ru(II) hydrides slowed significantly, decreasing by three orders of magnitude, down to 10^5^ and 10^4^ M^–1^ s^–1,^ for the ethyl and phenyl substituted complexes respectively, and by two (down to 10^8^ M^–1^ s^–1^) for the methyl substituted molecules.^54,55^ These rate constants appear to scale with respect to relative steric hindrance, with the most rapid kinetics achieved with dimethylphosphinoethane (dmpe). In the present work, we measured the bimolecular rate constant of the reaction occurring between photoexcited **1** (the Ru(0) intermediate) and phenylacetylene also using nanosecond transient absorption spectroscopy, Figure 3. Concentrations of phenylacetylene were systematically varied, and pseudo-first order kinetics were evaluated at 380 nm where there the transiently produced Ru(0) intermediate, the H_2_ elimination photoproduct, exhibits an absorption maximum in the difference spectrum.^16^ The second-order rate constant was calculated from the transient absorption kinetic data presented in Figure 3, yielding *k*_3_ = 7.47 ± 0.12 × 10^5^ M^–1^ s^–1^, which is the same order of magnitude as observed in the related Ru-based system introduced above. As these data echo one other, it is likely that the steric influence imparted by the triphenylphosphine ligands in **1** is largely responsible for the observed reaction rate constants of the Ru(0) intermediate with phenylacetylene. Overall, we believe this provides future opportunities for introducing unsaturated substrates that can effectively compete with H_2_ for the binding sites on the Ru(0) intermediate that can be leveraged in photocatalytic transformations.

Prepared with evidence for the likely coordination of phenylacetylene with photoactivated **1**, we explored the possibility of photoactivated hydrogenation by performing this reaction in the presence of H_2_. With **1**, H_2_ (3 atm), and phenylacetylene under continuous irradiation for 1 h at 365 nm, the turnover number (TON)^56^ was 4.16 for the formation of styrene and 0.145 for the generation of ethylbenzene in this photoreaction, Figure S5 and Table S3. This initial data is not that surprising given that reported long-term high temperature catalysis using a different substrate in the presence of **1** yielded a TON of 1.45 (one hydrogenation, cyclododecadiene) and 8.23 (two hydrogenations, cyclododecene).^45^ The shorter time scales involved selectivity, and room temperature conditions used here all lend credence to the photocatalytic activity of **1** as a hydrogenation catalyst. The photocatalysis is likely limited by both reformation of **1** with the available H_2_ in solution as well as established deleterious decomposition pathways in **1**.^14^ Even though **1** has clear limitations, it successfully provided the foundation for triplet photosensitized hydrogenation of unsaturated substrates. Echoing the *in situ* photochemical NMR experimental conditions above, substrates including *p*-tolylacetylene and ethylpropiolate also yielded photosensitized hyperpolarization in their hydrogenation products, Figures S13 and S14 and Table S1.The combined data illustrate our proof of concept of the Trip-PHIP process as a motif offering rapid and easily activated ^1^H NMR signal enhancement in model hydrogenation reactions.

### Transfer of Hyperpolarization of **1** to Substrates

**Figure 4:**
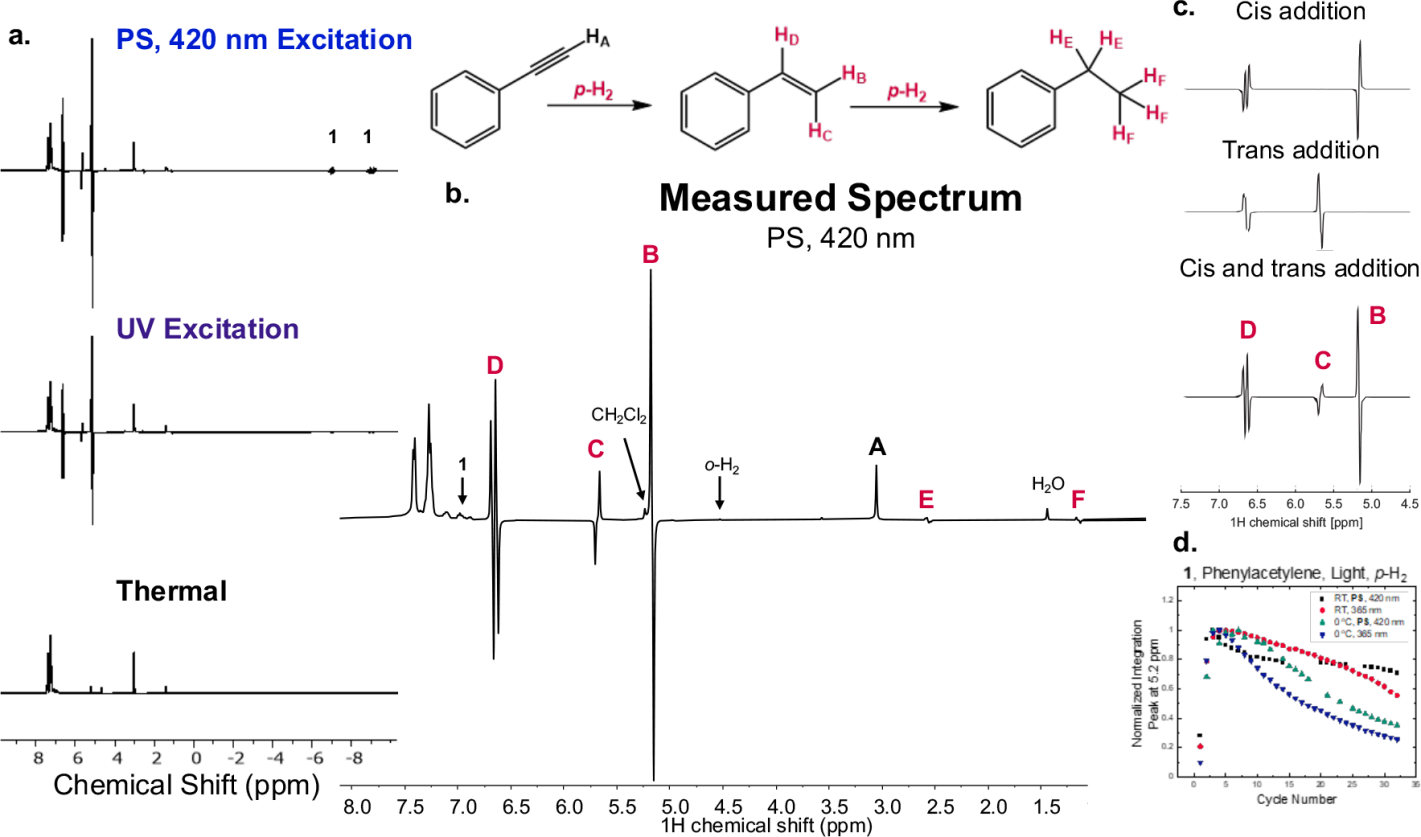
(a) ^1^H NMR spectra in deuterated dichloromethane, top to bottom of **1**, phenylacetylene, and **PS**, 420 nm light; **1**, phenylacetylene, and 365 nm light, and **1** and phenylacetylene in the dark. (b) ^1^H NMR spectrum of **1**, phenylacetylene, PS with 420 nm light and deuterated dichloromethane. (c) The simulated spectrum for hyperpolarized styrene in the absence of solvent, top to bottom of pure cis addition (H_D_ and H_B_), pure trans addition (H_D_ and H_C_), and 84% cis and 16% trans addition. (d) The normalized integrated signal of the peak at 5.2 ppm (H_B_) over a series of cycles with **1** and *p*-H_2_ as a function of direct excitation or photosensitization and as a function of the temperature (273 K (0 °C) or RT).

Armed with the knowledge that we can triplet photosensitize PHIP in **1** in the presence of *p*-H_2_ and that the photodriven hydrogenation of phenylacetylene to styrene (single hydrogenation) and ethylbenzene (double hydrogenation) is feasible, we explored these hydrogenation steps in the presence of 3 atm *p*-H_2_, to observe hyperpolarization in the hydrogenated substrates. Reactions with 3 atm *p*-H_2_, **1**, and phenylacetylene in the dark resulted in no substrate hydrogenation at RT, Figure 4a. However, 365 nm light stimulation of the same reaction mixture produces both styrene and ethylbenzene hydrogenation products featuring marked ^1^H NMR signal enhancement in the hydrogen atoms that were transferred from **1**, Figure 4b and Table S1. The 420 nm Ir(III) photosensitized reaction proceeds identically to the reaction using 365 nm direct excitation, featuring similar signal amplification in the ^1^H NMR spectrum of the styrene product, Table S2. Although phenylacetylene engages in an unknown sluggish quenching process with the **PS**, the rate constant for that reaction is several orders of magnitude below that of the reaction occurring between **1** and the **PS**, Figure S6, so it was not considered relevant. As anticipated, hyperpolarization was not observed in a sample of phenylacetylene and the **PS** in the dark, or with 420 nm light excitation if the **PS** was not present, Figures S7 and S8. When **1**, **PS**, and phenylacetylene were combined in the presence of 3 atm *p*-H_2_ and exposed to 420 nm light, hyperpolarized **1** was observed, but the ^1^H signal enhancement was significantly lower than that observed in the styrene hydrogenation product. The competition between the reformation of **1** with *p*-H_2_ and the association of the Ru(0) intermediate with phenylacetylene are evidenced in the hyperpolarization generated on both the hydrides in **1** and the hydrogenated products as shown in Figure 4a. In all instances, the ^1^H NMR signals from the hyperpolarized styrene dominates using either direct 365 nm in the absence of the **PS**, or 420 nm sensitized excitation in the presence of the **PS**. With repetitive cycling, the styrene signal increased with time, achieving a total enhancement of 906-fold in the direct (365 nm) and 1630-fold in the sensitized excitation (**PS** and 420 nm) of the catalyst, respectively, as compared to the thermal spectra after the experiment, Table S1. For the hydrogenated substrates, enhancement is the combination of the integral of a hyperpolarized peak for all the cycles of a run compared to the thermal spectrum taken after the cycles of light and *p*-H_2_, after the polarization decayed. This calculation combines the hyperpolarization over cycles to account for the total reaction and all product formed. Even though this model reaction is inefficient as compared to other catalytic reactions,^57^ photoactivated **1** yielded substantial enhancement in the hydrogenation of the substrate with *p*-H_2_. Clearly, Ru(CO)(PPh_3_)_3_(H)_2_ can serve as a photocatalyst for transferring PHIP into unsaturated substrates at high field, under both direct and triplet sensitized reaction conditions.

Although we have demonstrated light-driven hyperpolarization in a range of *para*-hydrogenated substrates, the most efficient signal enhancement was derived from the reactions with phenylacetylene. As discussed earlier, **1** is known to catalytically hydrogenate unsaturated substrates at elevated temperatures; however, lower temperature activation is more desirable if it can be accessed. Accordingly, we also tested the photocatalytic hydrogenation of phenylacetylene at reduced temperature of 273 K (0 °C) and monitored the Trip-PHIP reaction, Figure 4d. Compared to the experiments at RT described above, similar polarized NMR enhancements of the styrene hydrogenation product are obtained at 0 °C using direct and photosensitized excitation, resulting in total 447- and 226-fold increases with respect to the thermally equilibrated samples measured after the reaction, respectively, Figure S18 and Table S2. We believe this result is particularly intriguing as the photoinduced reaction yielded similar NMR signal amplification with respect to the RT samples and illustrates the power of photoactivation applied to these model hydrogenation reactions and the resultant hyperpolarization that can be achieved.

### Simulation of the Hyperpolarized Styrene Spectral Features

Figure 4c presents the simulated ^1^H NMR spectrum of hyperpolarized styrene. The hyperpolarized spin system in styrene consists of three spins as indicated in Figure 4. We used the freeware Spinach^58^ to reproduce the spectral features and to quantify the relative contribution of *cis*- and *trans*-addition to the phenylacetylene. First, we confirmed by simulation that initiation in a *p-*H_2_ induced singlet state on the spin pair H_a_, H_b_ (**I**_a_·**S**_b_ = *I*_xD_·*I*_xD_ + *I*_yD_·*I*_yB_ + *I*_zB_·*I*_zB_) quickly turns into *I*_zD_·*I*_zB_ due to the incoherent nature of the hydrogenation and fast dephasing of the transverse (*x*,*y*) components at high magnetic field. This implies that a pure *cis*-addition would only give hyperpolarized signals corresponding to H_D_ and H_B_. However, in the experimental spectrum, hyperpolarized H_c_ is also observed. This implies that there is either some portion of *trans*-addition, or *cis*-addition followed by fast *cis*-*trans* isomerization such that some *I*_zD_·*I*_zC_ spin order is also generated through hydrogenation with *p*-H_2_ and incoherent averaging. We note that on the time scale of the NMR experiment, *trans*-addition and *cis*-addition followed by fast *cis*-*trans* isomerization are indistinguishable, and we will refer to these two simply as *trans*-addition. In other words, *cis*-addition generates *I*_zD_·*I*_zB_ spin order, and *trans*-addition generates *I*_zD_·*I*_zC_ spin order as depicted in Figure 4. To quantify the relative contribution of the two mechanisms, we varied the relative contributions of each and found that an initial density matrix of 0.84 *I*_zD_·*I*_zB_ + 0.16 *I*_zD_·*I*_zC_ gave a good match with the experimental result indicating that about 16% of the hydrogenation results in a *trans* fashion, while the majority occurs through the expected *cis*-addition, Figure 4c. By simulating the hyperpolarization of styrene, we gained additional insights into the mechanism by which **1** likely hydrogenates phenylacetylene.

### Hyperpolarization of Styrene via Exchange

We observed an additional mechanism for the formation of hyperpolarized styrene via exchange. Instead of *para*-hydrogenation of phenylacetylene as the exclusive pathway leading to hyperpolarized styrene, we also observed that hyperpolarized styrene can be generated from styrene itself, through an exchange process. Experiments performed with *p*-H_2_, **1**, styrene, using either 365 nm or photosensitization by the PS at 420 nm, produced hyperpolarized styrene in addition to the expected hyperpolarized ethylbenzene, Figures S12 and S13. Please note that if hydrogenation was the only hyperpolarization mechanism, styrene should not become hyperpolarized in these experiments. The generation of hyperpolarization on styrene indicates an exchange between the hydrides of **1** and the alkene protons. We note that this process produces polarization levels on the same order as thermal polarization, Figures S12 and S13. Similar exchange processes have been demonstrated previously with **1**, where a cross-coupling reaction involving both acetophenone-*d*_5_ and vinyltrimethylsilane formed a cross-coupling product and demonstrated H/D exchange with both styrene and vinyltrimethysilane;^24,44^ our subsequent experiments echoed these results. TON experiments were performed with **1**, H_2_ (3 atm), 60% styrene-*d*_3_, and 1 h of 365 nm irradiation.^59,60^ This resulted in the formation of ethylbenzene, a TON of 2.81, and an increase in styrene-*d*_*n*_ (*n* < 3) having a TON of 1.78 (Table S4), indicating the exchange of protons from **1**. Extrapolating to our model reaction with phenylacetylene, there are likely two mechanisms simultaneously occurring leading to the observation of hyperpolarization in the reaction products. The dominant process is the hydrogenation of the triple bond by **1** (which generated 200–1630-fold signal enhancements, Tables S1 and S2) and a minor pathway featuring proton exchange of **1** with styrene, generating signals only slightly above thermal polarization. Experiments with styrene as a reactant indicated that the polarization occurring through exchange, as measured by the hyperpolarization on styrene, decreased with cycle number, possibly due both to the consumption of styrene and the decrease of exchange. Specifically, these experiments using styrene featured antiphase ^1^H NMR signals which were only visible up to cycle 6 (**PS**, 420 nm), or cycle 3 (365 nm). Small hyperpolarization effects were still produced on longer time scales as demonstrated by the differences between the thermal spectra and the spectra from cycles under light activation, Figures S12 and S13. The exchange induced polarization is similar in magnitude to thermal polarization as evident in Figures S12 and S13 and Table S1, and the antiphase signals are present in fewer cycles (up to cycle 3 or 6) for exchange as compared to the antiphase signals following hydrogenation (cycle 32). We conclude that the antiphase signals, and much of the hyperpolarization observed with phenylacetylene as the substrate, originate primarily from direct hydrogenation by **1** and do not result from proton exchange.

## Conclusions

We have demonstrated triplet photosensitized *para*-hydrogen induced polarization (Trip-PHIP) using visible light excitation for the first time. ^1^H NMR hyperpolarization was generated on the hydride ligands of Ru(CO)(PPh_3_)_3_(H)_2_ as well as on hydrogenated organic substrates, using either 365 nm light for direct excitation, or 420 nm light in conjunction with an Ir(III) photosensitizer. The hydrogenation-based photoreactivity of the ruthenium catalyst **1** enabled intimate control of the hyperpolarization realized in the target substrates. To the best of our knowledge, the Trip-PHIP NMR signal enhancements, 1630-fold from a sample of **1**, **PS**, and phenylacetylene, measured here represent the highest reported PHIP-derived hyperpolarization generated through photoactivated hydrogenation of an organic substrate. In these photochemical-based experiments, signals were generated more rapidly than traditional PHIP approaches, achieving hyperpolarized resonances after only 0.8 s of LED irradiation. Trip-PHIP represents a facile means for generating light-triggered hyperpolarization, gaining valuable mechanistic insight of potentially tremendous value in chemical catalysis and providing the foundation for future studies having relevance to light-activated hyperpolarized MRI contrast agents and tracers.

## Supporting Information Available

Complete Experimental Section, general experimental procedures, static and transient absorbance spectra, static photoluminescence spectra, ^1^H and ^31^P NMR spectra, including ^1^H NMR spectra with *p*-H_2_ and enhancement calculations, the electronic structure calculations performed, and details regarding the *in situ* pulse program and NMR tube construction (PDF)

## Abbreviations

NMR: nuclear magnetic resonance
PHIP: *para*-hydrogen induced polarization
*p*-H_2_: *para*-hydrogen
*o*-H_2_: *ortho*-hydrogen
PPh_3_: triphenylphosphine
Ru(CO)(PPh_3_)_3_(H)_2_: **1**
Trip-PHIP: triplet sensitized PHIP
PS: photosensitizer
RT: room temperature
DCM: dichloromethane.
