# Supplementary material for: Triplet Photosensitized para-Hydrogen Induced Polarization
Source: ACS Cent Sci. 2022 Nov 14;8(11):1548–56. doi: 10.1021/acscentsci.2c01003 (PMC9686209; doi:10.1021/acscentsci.2c01003)
Supplement: Supplementary file 1 — oc2c01003_si_001.pdf [file oc2c01003_si_001.pdf]

Supporting Information for:

Triplet Photosensitized Para-Hydrogen Induced Polarization

Emily E. Brown, Iuliia Mandzhieva, Patrick M. TomHon, Thomas Theis, and Felix N. Castellano**

Department of Chemistry, North Carolina State University, Raleigh, NC, 27965-8204

E-mail: fncastel@ncsu.edu & ttheis@ncsu.edu

Table of Contents

General Experimental **S2**

Estimation of Ligand-field Triplet State Energies **S3**

Quenching Studies **S17**

Static Photoluminescence Measurements **S17**

Nanosecond Transient Absorbance and Photoluminescence Measurements **S17**

Transient Absorption of 1 and PS **S18**

^{1}H NMR Spectra of Continuous Irradiation of 1 and PS **S19**

***In-Situ* NMR Measurements** **S19**

***In-Situ* Pulse Program and NMR Tube** **S20**

In-Situ NMR **Tube** **S20**

Pulse **Program** **S20**

In-Situ Bubbling of p-H_2 .... **S20**

In-Situ **Irradiation** S21

Pulse Program **Triggers** S21

^{1}H NMR **Acquisition** S21

Hyperpolarization Enhancement **Calculations** S21

Quantitative NMR Measurements S24

Phenylacetylene and **1, TON** S24

Deuterated Styrene and **1, TON** S25

Quenching of PS by Organic Substrates S26

Quenching Rate of PS by Organic **Substrates** S27

^{1}H NMR Spectra S27

Nuclear Spin Simulations S28

^{1}H NMR Spectra of Styrene Exchange S31

T_1 of Styrene S33

^{1}H NMR Spectra with Selected Organic Substrates S34

Hyperpolarization at 0 °C S36

References S36

General Experimental. The synthesis and structural characterization of **1** echoed previous literature.[1] All solvents were aerated, unless otherwise specified, and used as received. The p-H_2 gas was generated at ~ 94 % enrichment using an Advanced Research Systems p-H_2 generator with a ARS-4HW compression and a Lakeshore Model 335 Cryogenic Temperature Controller.[2,3] Absorbance spectra were measured using an Agilent Cary 60 UV-Vis spectrometer, unless otherwise specified.

Estimation of the Ligand-field Triplet State Energies.

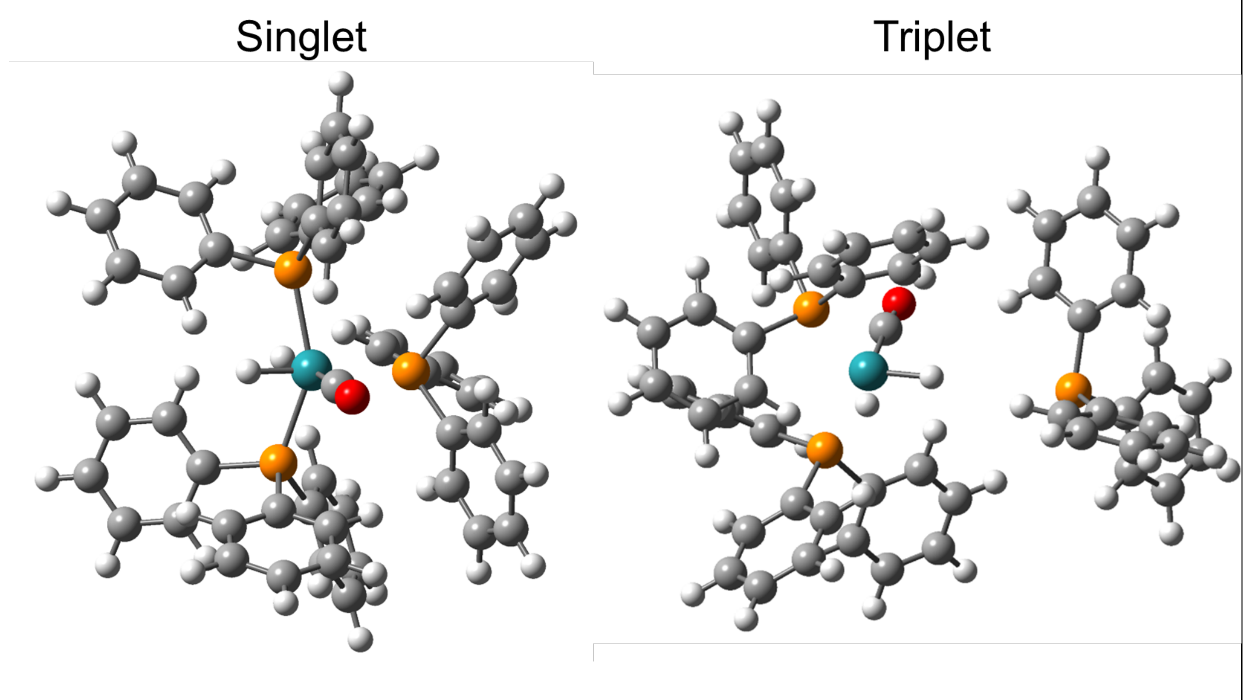

Figure S1. Optimized geometries of $Ru(CO)(PPh_3)_3(H)_2$, left to right, in the singlet state and the first excited triplet state.

DFT calculations, using Gaussian 16, were preformed to estimate the triplet energy of **1**.[4] The singlet and triplet geometries were optimized using the B3LYP functional.[5] The LANL2DZ[6–9] basis set was used for the ruthenium center and the basis set 6-31G**[10–18] was used for the H, C, O, P atoms. Calculations were performed without solvation and with the keywords “gfinput” “scf=(conver=8,maxcycle=500)” and “pop=full”.

XYZ coordinates of atoms in the optimized structure of $^1Ru(CO)(PPh_3)_3(H)_2$

Ru	0.01430	0.55080	-0.37160
H	0.04680	2.13940	-0.58530

H	-0.04430	0.85620	1.27120
C	0.14620	0.36340	-2.28700
O	0.24420	0.30170	-3.44460
P	0.03800	-1.94790	-0.05250
P	-2.31190	1.02990	-0.00340
P	2.28620	1.15570	-0.01710
C	3.44850	0.06920	0.94890
C	2.99510	-0.40570	2.18990
C	4.74770	-0.26090	0.53690
C	3.81300	-1.19960	2.99320
H	1.99170	-0.15990	2.52010
C	5.56680	-1.05770	1.34200
H	5.12490	0.09670	-0.41470
C	5.10220	-1.53220	2.56920
H	3.43630	-1.56660	3.94350
H	6.57050	-1.30470	1.00630

H	5.73920	-2.15470	3.19130
C	2.54060	2.72950	0.95010
C	1.46270	3.51000	1.38420
C	3.84430	3.14480	1.27690
C	1.67930	4.68120	2.11590
H	0.45420	3.19120	1.15300
C	4.06050	4.31480	2.00290
H	4.69720	2.54960	0.96620
C	2.97600	5.08840	2.42530
H	0.82730	5.27120	2.44220
H	5.07550	4.61910	2.24300
H	3.14400	5.99840	2.99480
C	3.21620	1.48520	-1.58650
C	3.48960	0.40700	-2.44660
C	3.56830	2.77800	-1.99980
C	4.12490	0.61520	-3.66980

H	3.20250	-0.60100	-2.16310
C	4.19070	2.98580	-3.23360
H	3.35900	3.62870	-1.36100
C	4.47730	1.90660	-4.06860
H	4.33290	-0.23340	-4.31520
H	4.45240	3.99570	-3.53740
H	4.96420	2.06960	-5.02600
C	-3.03070	0.15260	1.46570
C	-3.74210	-1.04970	1.33690
C	-2.75810	0.63070	2.75990
C	-4.18820	-1.74000	2.46730
H	-3.95220	-1.45700	0.35450
C	-3.21360	-0.05330	3.88640
H	-2.18800	1.54530	2.88720
C	-3.93320	-1.24250	3.74430
H	-4.73470	-2.67020	2.34110

H	-3.00380	0.34360	4.87610
H	-4.28620	-1.77720	4.62150
C	-3.61720	0.80500	-1.32460
C	-3.22960	0.85530	-2.67050
C	-4.98710	0.70290	-1.03030
C	-4.17730	0.78470	-3.69470
H	-2.18150	0.95110	-2.92610
C	-5.93370	0.62250	-2.05160
H	-5.32300	0.68090	0.00060
C	-5.53240	0.66080	-3.38870
H	-3.85100	0.82660	-4.73010
H	-6.98740	0.53530	-1.80060
H	-6.27090	0.60110	-4.18320
C	-2.70650	2.82220	0.37940
C	-3.79970	3.18170	1.18590
C	-1.98600	3.84730	-0.24990

C	-4.14000	4.52110	1.37870
H	-4.39030	2.41680	1.67740
C	-2.33170	5.18780	-0.06540
H	-1.14400	3.59230	-0.88430
C	-3.40600	5.53110	0.75500
H	-4.98320	4.77280	2.01620
H	-1.75480	5.96190	-0.56400
H	-3.67160	6.57380	0.90480
C	1.35830	-2.80310	-1.05730
C	1.11030	-3.21460	-2.37660
C	2.65920	-2.95710	-0.54810
C	2.12710	-3.76280	-3.16040
H	0.11710	-3.11850	-2.80040
C	3.67350	-3.51220	-1.33100
H	2.88730	-2.65040	0.46570
C	3.41330	-3.91590	-2.64130

H	1.90740	-4.07540	-4.17750
H	4.66890	-3.62220	-0.91010
H	4.20290	-4.34760	-3.24990
C	-1.48050	-2.86490	-0.64110
C	-1.99260	-4.00500	-0.00230
C	-2.14840	-2.38700	-1.77980
C	-3.13300	-4.64580	-0.49080
H	-1.51130	-4.39320	0.88750
C	-3.28410	-3.03130	-2.27420
H	-1.78910	-1.49380	-2.27610
C	-3.78250	-4.16360	-1.62850
H	-3.51430	-5.52410	0.02330
H	-3.78480	-2.63240	-3.15160
H	-4.67130	-4.66240	-2.00470
C	0.28180	-2.68380	1.63450
C	-0.12650	-1.93340	2.74560

C	0.80480	-3.97090	1.84900
C	-0.01500	-2.45120	4.03770
H	-0.52350	-0.93590	2.59150
C	0.92550	-4.48450	3.14110
H	1.12620	-4.57390	1.00630
C	0.51570	-3.72580	4.23990
H	-0.34330	-1.85410	4.88380
H	1.33860	-5.47880	3.28780
H	0.60910	-4.12730	5.24520

XYZ Coordinates for the optimized geometry of $^3Ru(CO)(PPh_3)_3(H)_2$

Ru	-0.86080	0.15390	0.10750
H	0.45630	0.59140	0.96670
H	-1.26090	0.00870	1.69600
C	-0.06140	0.48560	-1.65550
O	0.48010	0.71460	-2.65560
P	-1.37610	-2.21680	-0.06830

P	-2.74240	1.68880	0.14760
P	4.54740	0.09510	-0.11910
C	6.20740	-0.59610	-0.56870
C	6.52820	-1.87810	-0.08380
C	7.11140	0.03060	-1.43880
C	7.72280	-2.50170	-0.43950
H	5.83840	-2.38860	0.58440
C	8.30340	-0.60060	-1.80550
H	6.88690	1.01660	-1.83190
C	8.61550	-1.86470	-1.30590
H	7.95470	-3.48760	-0.04560
H	8.99000	-0.09770	-2.48130
H	9.54360	-2.35240	-1.59040
C	4.73360	0.43540	1.69340
C	3.54690	0.60630	2.42680
C	5.96190	0.52110	2.36790

C	3.58980	0.87610	3.79530
H	2.58470	0.52510	1.92570
C	6.00210	0.78120	3.73940
H	6.88940	0.37590	1.82260
C	4.81740	0.96270	4.45540
H	2.66200	1.00820	4.34510
H	6.96080	0.84280	4.24780
H	4.85080	1.16410	5.52260
C	4.58610	1.79780	-0.84860
C	3.78910	2.03030	-1.98060
C	5.34060	2.86090	-0.32520
C	3.75860	3.28930	-2.58530
H	3.17550	1.22860	-2.38130
C	5.30780	4.11880	-0.92720
H	5.94750	2.70600	0.56180
C	4.51860	4.33490	-2.06020

H	3.13320	3.45150	-3.45860
H	5.89550	4.93220	-0.50990
H	4.49120	5.31650	-2.52540
C	-4.35780	1.02520	0.76260
C	-5.58970	1.39880	0.20150
C	-4.34720	0.10560	1.82490
C	-6.78260	0.86590	0.69560
H	-5.62010	2.10480	-0.62160
C	-5.54090	-0.42160	2.31780
H	-3.39760	-0.20340	2.24880
C	-6.76180	-0.04440	1.75350
H	-7.72800	1.16370	0.25040
H	-5.51190	-1.13740	3.13420
H	-7.69080	-0.46090	2.13270
C	-3.20740	2.49620	-1.45180
C	-3.27580	1.69260	-2.60210

C	-3.48360	3.86690	-1.56630
C	-3.62390	2.24480	-3.83400
H	-3.05060	0.63210	-2.53350
C	-3.82160	4.41980	-2.80400
H	-3.43180	4.50590	-0.69110
C	-3.89460	3.61130	-3.93880
H	-3.67260	1.60890	-4.71330
H	-4.02590	5.48420	-2.87860
H	-4.15500	4.04370	-4.90070
C	-2.39430	3.11630	1.26650
C	-3.38090	3.70850	2.06880
C	-1.08650	3.62630	1.30570
C	-3.06530	4.79330	2.88980
H	-4.39510	3.32250	2.05700
C	-0.77620	4.71350	2.12220
H	-0.30910	3.15810	0.70850

C	-1.76450	5.29860	2.91690
H	-3.83750	5.24070	3.50940
H	0.24020	5.09570	2.14380
H	-1.52060	6.14080	3.55830
C	0.11230	-3.28940	-0.31480
C	0.02050	-4.69150	-0.28060
C	1.36290	-2.69510	-0.53470
C	1.15370	-5.47810	-0.47980
H	-0.93570	-5.16990	-0.09300
C	2.49780	-3.48570	-0.73280
H	1.45810	-1.61370	-0.54100
C	2.39450	-4.87600	-0.70860
H	1.06910	-6.56100	-0.45240
H	3.45610	-3.00360	-0.90060
H	3.27680	-5.49140	-0.86170
C	-2.43930	-2.57350	-1.53660

C	-3.78310	-2.15530	-1.52340
C	-1.92100	-3.14090	-2.71050
C	-4.58830	-2.32010	-2.64980
H	-4.20260	-1.70410	-0.62900
C	-2.72850	-3.29470	-3.83980
H	-0.88660	-3.46570	-2.74490
C	-4.06300	-2.88900	-3.81330
H	-5.62560	-1.99890	-2.61840
H	-2.31060	-3.73690	-4.73990
H	-4.68970	-3.01300	-4.69180
C	-2.24440	-3.07010	1.32770
C	-1.88100	-2.72720	2.64000
C	-3.20710	-4.07270	1.13280
C	-2.46370	-3.37460	3.72950
H	-1.15360	-1.93720	2.80020
C	-3.79570	-4.71310	2.22590

H	-3.50790	-4.35100	0.12820
C	-3.42530	-4.36730	3.52590
H	-2.16990	-3.09810	4.73820
H	-4.54380	-5.48300	2.05780
H	-3.88330	-4.86680	4.37510

Quenching Studies. Stern Volmer measurements monitor the decrease in the photosensitizer's photoluminescence and lifetime as a function of increasing concentration of the quencher, here **1**.[19] Once plotted, this enables calculation of the slope which yields the rate of quenching. Stern Volmer constants were generated from linear fits of photoluminescent data calculated from Origin 2021b software. The uncertainty in the fit slopes were assumed to dominate the uncertainty in the resulting k_q values. Quenching studies were preformed using nanosecond photoluminescence kinetic measurements as described, *vide infra*, and using static photoluminescence measurements, *vide infra*.

Static Photoluminescence Measurements. Samples were prepared in aerated spectroscopic grade dichloromethane. Measurements were performed using an Edinburgh Instruments FS920 photoluminescence spectrometer. Samples were excited at 420 nm, averaged three times, and were corrected using the correction files on the FS920 software. Integration and integrals were calculated using Origin 2021b software.

Nanosecond Transient Absorbance and Photoluminescence Measurements. Measurements were performed using an Edinburgh Instruments LP920 spectrometer which employed a tunable OPO laser (Opotek Vibrant 355). Samples were prepared in aerated spectroscopic grade

dichloromethane and were excited with a pulse of 1 Hz. Lifetimes were calculated using Origin 2021b single exponential decay. Nanosecond photoluminescence kinetic measurements were performed with excitation pulses of 420 nm (1.5 mJ), and were monitored at 470 nm, with a bandwidth of 1 nm, and 50 averages. The absorbance of the **PS** solution was 0.32 OD at 420 nm. Nanosecond absorbance kinetic measurements, unrelated to the quenching studies, were performed with excitation pulses of 355 nm (1 mJ), and were monitored at 380 nm, with a bandwidth of 4 nm and 100 averages unless otherwise specified. The absorbance of starting solution was 0.78 OD at 355 nm. The resulting absorbance kinetic data was smoothed with the Savitzky-Golay formula, 30 points and second order polynomial.

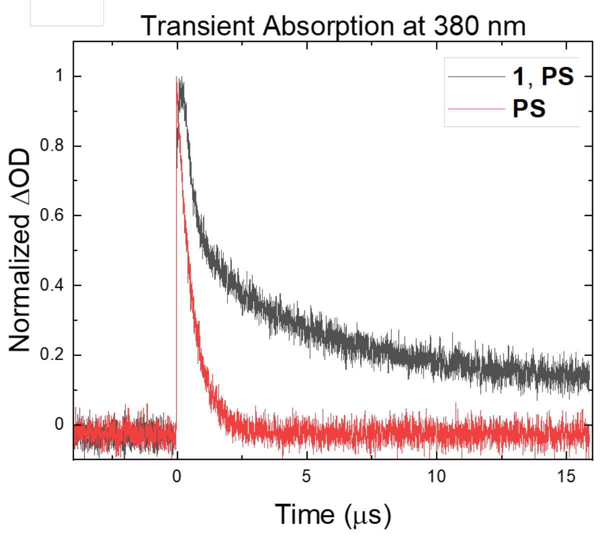

Figure S2. Nanosecond transient absorption of **PS** with and without **1**, exciting at 420 nm (1 mJ) and monitoring 380 nm where the photosensitized ruthenium complex rebinds with H_2. Used a 380 nm bandpass filter placed before the detector.

Nanosecond transient absorption, above, performed with 420 nm excitation pulses of 1 mJ, and 100 averages. Used aerated DCM, and 380 nm bandpass filter before the detector. Absorbance of **PS** for both samples: 0.26 OD at 420 nm.

^{1}H NMR Spectra of Continuous Irradiation of 1 and PS.

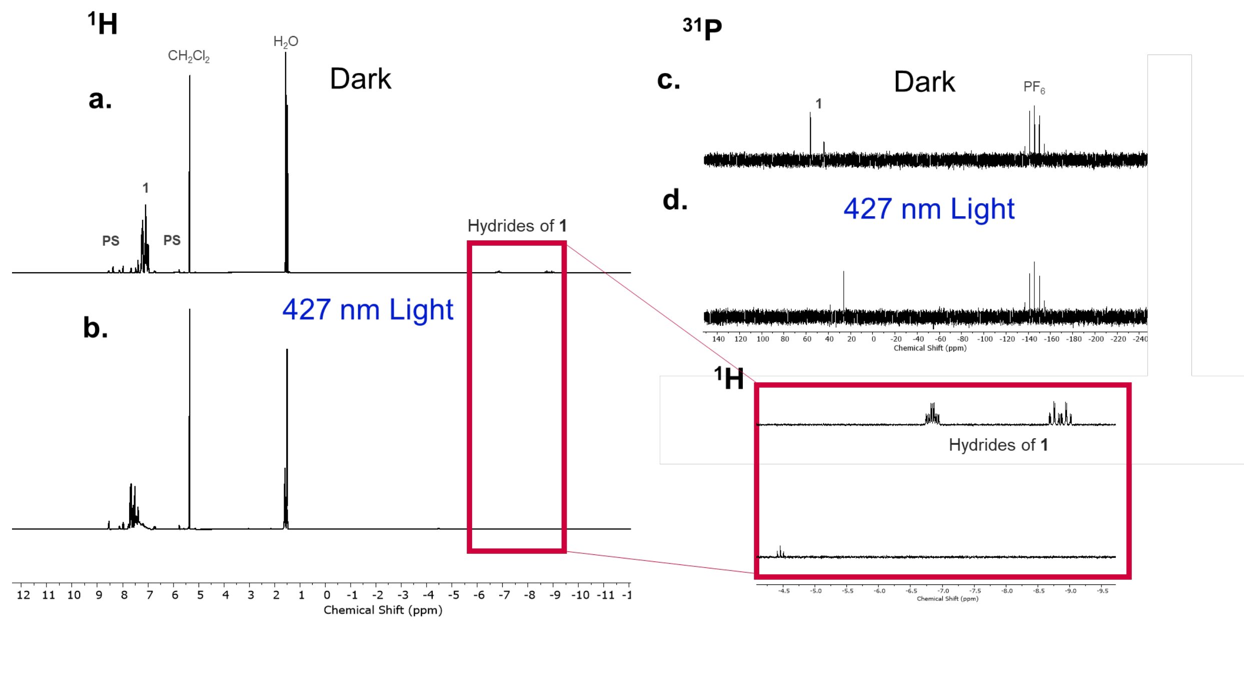

Figure S3. ^{1}H and ^{31}P NMR spectra of **1** and **PS**, in the dark, or after 5 min of 427 nm irradiation with a Kessil Lamp on the highest setting. Irradiated sample inside the NMR tube.

For the constant irradiation of the samples, a 427 nm Kessil Lamp at the highest setting was used (~ 150 mW cm^{-1}), approximately 2 inches away from sample, with the constant use of a fan.

***In-Situ* NMR Measurements.** The pulse program used here on the 400 MHz Bruker NEO is described in detail in the SI. Briefly, the *p*-H_2 was bubbled through the sample for 20 s, followed by a one second waiting time, the light was turned on for 0.8 s prior to the application of a 45° pulse, and then the spectrum was acquired. After the acquisition of the spectrum was complete,

the light was turned off. The cycle then looped back and repeated. Each spectrum was measured before and after the cycles of both light excitation and p-H_2 bubbling for the thermal comparison and enhancement calculations. The p-H_2 was maintained at 50 psi with a flow rate of 45 sccm. The light was 365 nm (63 mW) or 420 nm (63 mW), and was shone through a fiber optic cable, inside an inner glass capillary tube to irradiate the NMR sample *in-situ* (Figure S4). All ^{1}H NMR spectra, unless otherwise specified, consisted of a single scan.

Sample concentrations were approximately: ~ 0.5 mL DCM-d$_2$, ~ 2-3 mM **1**, ~ 0.9-1 mM **PS**, and ~ 0.3 M phenylacetylene or ~ 0.4 M ethylpropiolate.

***In-Situ* Pulse Program and NMR Tube.**

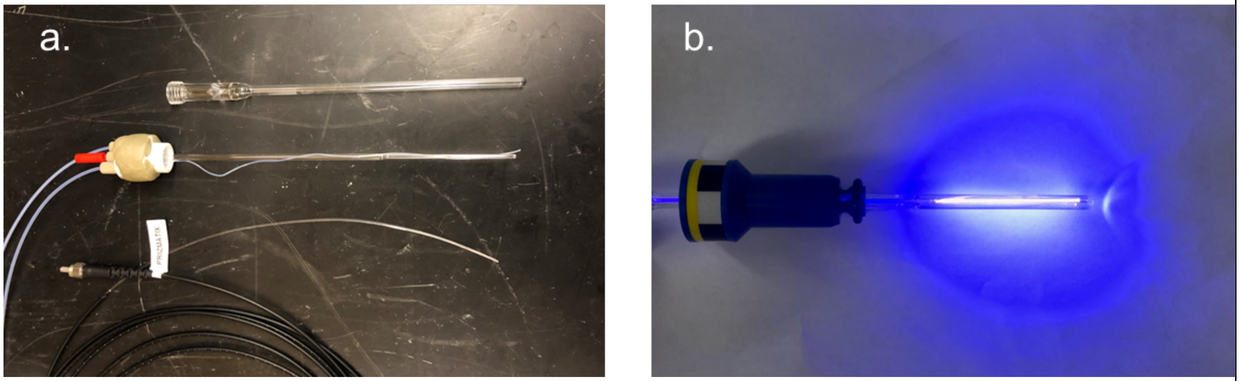

Figure S4. a. Top to bottom, *in-situ* NMR tube; cap with coaxial tube, capillary for p-H_2 bubbling, and inflow and outflow tubing; fiberoptic cable with sanded end; b. *in-situ* NMR tube in action, if not physically *in situ*.

Pulse Program. Samples were measured using a Bruker Ascend 400 MHz with a NEO Avance console and all measurements are one scan unless specified otherwise. *Para*-Hydrogen at ~94% purity and generated an Advanced Research Systems p-H_2 generator with a ARS-4HW compressor and a Lakeshore Model 335 Cryogenic Temperature Controller.[2,3]

Bubbling parameters: 50 psi, 45 sccm flow rate. All samples in aerated dichloromethane-d_2.

***In-Situ* Bubbling of *p*-H_2.** We used the New Era NMR tube: NE-CAV5-G-140 and coaxial tube: NE-379-5-COIC. The cap was custom made, with an inflow capillary tube of Teflon, outflow tubing, and a seal around the coaxial tube. The cap is rated for 50 psi and primarily made of Teflon, except for an O-ring, IDEX connectors for the inflow and outflow tubing and the coaxial tube, and epoxy sealant.

***In-Situ* Irradiation**. A Prizmatixs FC5-LED multi-channel device was coupled to an NMR fiber to irradiate the sample *in situ*. The fiber was a high NA polymer optical fiber (POF), 4 m long with the last 23 cm stripped of cladding and the final 5 cm sanded for irradiation. The core diameter is 1000 µm. The LEDs used here were 365 nm, with a maximum power of 70 mW, and 420 nm, with a maximum power of 100 mW.

Pulse Program Triggers. A TTL pulse was applied from the Bruker 400 MHz NEO console to an inversion circuit which was used to turn on the light, as described in the pulse program. The inversion circuit used a PNP transistor: A1015 GR 331 with a collector-emitter and collector-base voltage of -50 V, and used two resistors, one 150 kΩ ± 5% and one 15 kΩ ± 5%. The inversion circuit was connected to a BK Precision 180 W Multi Range 60V/8 A DC Power Supply which supplied about 3 V to the circuit. The *p*-H_2 bubbling, as described in the pulse program, was also triggered using a TTL pulse.[20] The outflow was regulated using FlowDDE software, version 3.35 and a Bronkhorst EL-FLOW Select F-201CV mass flow controller for gases.

^{1}H NMR Acquisition. The parameters for the one scan spectra are as follows: Time points: 130718, Sweep Width: 32679.75 Hz, 81.6728 ppm; Acquisition: 1.9999855 s; Receiver gain: 1, Dwell time: 15.3 µs, Pre-Scan Delay: 50 µs, Delay after bubbling: 0.00001 s, Irradiation before pulse: 0.8 s. Pulse: 5 µs, 16.387 W, -12.14 dB.

Hyperpolarization Enhancement Calculations. To calculate the enhancement, the signals of a specified peak, measured over the course of series of cycles, were individually integrated and converted to the magnitude mode of Topspin 4.1.3, then were summed with the other peak values of that same series of cycles, and compared to a thermally polarized spectra. For measurements of the hydride peaks of **1**, the thermally polarized spectra were taken before the cycles of light and *p*-H_2 and for measurements of the hydrogenated products, the thermal spectra were taken after the cycles of light and *p*-H_2. In all cases, the thermal spectra were measured with the same parameters as mentioned in the previous section and were only one scan. The hyperpolarized peaks intensities were summed to account for the full scale of the reaction, and individual peak enhancements have also been included. The individual peak enhancements represent the highest signal enhancement of a given series of cycles and are compared to the thermally polarized spectra. Occasionally, measurements were discarded as they did not phase properly, which have been interpreted as bubbles remaining in the probe. To account for this, an average signal enhancement value for each series of spectra has also been included.

Table S1. Hyperpolarization at RT

Substrates	Excitation (nm)	Species with Highest Hyperpolarization	Enhancement over Series of Cycles	Enhancement of Highest Single Scan	Average Enhancement
**1**	365	**1**	35.6	13.6	1.11
**1**	420, **PS**	**1**	235	71.2	7.34

**1**, Phenylacetylene	365	Styrene	906	83.8	28.3
**1**, Phenylacetylene	420, **PS**	Styrene	1630	83.1	65.1
**1**, Ethylpropiolate	365[1]	Ethylacrylate	44.9	9.84	2.81
**1**, Ethylpropiolate	420,[1] **PS**	Ethylacrylate	6.36	5.82	1.59
**1**, p-Tolylacetylene	365	4-Methylstyrene	302	28.6	14.4
**1**, p-Tolylacetylene	420, **PS**	4-Methylstyrene	39.7	27.7	5.67
**1**, Styrene	365	Styrene	13.5	1.34	0.422
**1**, Styrene	420, **PS**	Styrene	24.8	1.46	0.802

[1] Continuous light irradiation

Table S2. Hyperpolarization at 0°C

Substrates	Excitation (nm)	Species with Highest Hyperpolarization	Enhancement over Series of Cycles	Enhancement of Highest Single Scan	Average Enhancement

**1**	365	**1**	50.3	127	1.80
**1**	420, **PS**	**1**	380.	156	15.8
**1**, Phenylacetylene	365	Styrene	447	95.9	14.0
**1**, Phenylacetylene	420, **PS**	Styrene	226	54.8	8.69

Quantitative NMR Measurements. Three samples were prepared of 0.2 mM of **1**, 0.2 M phenylacetylene in DCM-d_2. Spectroscopic grade toluene used as a standard. The pressure of H_2 was set to 50 psi, 45 sccm (cm^3/min) outflow. Samples run in usual *in-situ* irradiation NMR system, although they were not physically *in situ* of the NMR. H_2 was bubbled into sample for two minutes, after which the samples were irradiated with 365 nm (70 mW) light in the NMR tube for 1 hour. After irradiation, samples were depressurized and promptly measured. Samples were measured with 32 scans with 30 s delays between acquisitions.

Quantitative NMR and TON.

Table S3. Phenylacetylene and 1, TON

Sample	Styrene, dark (M)	Ethylbenzene, dark (M)	Styrene, after light (M)	Ethylbenzene, after light (M)	**1**, dark (M)	TON Styrene	TON Ethylbenzene
1	0.000986	0.000719	0.01470	0.00138	0.00377	3.67	0.149
2	0.00113	0.000815	0.0133	0.00133	0.00252	4.91	0.117
3	0.00165	0.00119	0.0157	0.00188	0.00367	3.89	0.170

All concentrations calculated from peak integrals, as compared to calculated sample volume. Averaged styrene concentrations from integrals of peaks at 6.891—6.750 ppm (H_D) and 5.942—5.755 ppm (H_c), ethylbenzene from integrals of peaks at 2.785—2.676 ppm (H_E) and 1.357—1.294 ppm (H_F), divided by total protons per peak, and **1** from integrals -6.441— -7.049 ppm and -8.503 — -8.998 ppm. Calculated standard toluene concentration assumed to be proportional to the ratio of peak integrals of toluene to DCM before irradiation. That constant was found for the known concentration of toluene pre irradiation and assumed to apply for the sample post irradiation.

Table S4. Deuterated Styrene and 1, TON

Sample	Styrene, dark (M)	Ethylbenzene, dark (M)	Styrene, after light (M)	Ethylbenzene, after light (M)	**1**, dark (M)	TON Styrene	TON Ethylbenzene
1	0.00799	0.000495	0.00972	0.00234	0.00325	2.44	2.63
2	0.0279	0.00131	0.0294	0.00865	0.00205	1.06	3.90
3	0.0193	0.00138	0.0261	0.00987	0.0126	1.85	1.90

All concentrations calculated from peak integrals, as compared to calculated sample volume. Styrene comprised of 60.05% styrene-d_3 at H_D, H_C and H_B, and was synthesized using the catalyst $Ru(CO)(PPh_3)_3HCl$.[1,21] Averaged styrene (here styrene-d_n ($n \leq 3$)) concentrations from integrals of peaks at 6.891—6.750 ppm (H_D) and 5.942—5.755 ppm (H_c), ethylbenzene (here ethylbenzene-d_n ($n \leq 3$)) from integrals of peaks at 2.785—2.676 ppm (H_E) and 1.357—1.294 ppm (H_F), divided by total protons per peak, and **1** from integrals -6.441— -7.049 ppm and -8.503 — -8.998 ppm. Calculated standard toluene concentration assumed to be proportional to the ratio of peak integrals of toluene to DCM before irradiation. That constant was found for the known concentration of

toluene pre irradiation and assumed to apply for the sample post irradiation. Growth of styrene peaks post irradiation assumed to be an increase in styrene-d_l ($l < 3$).

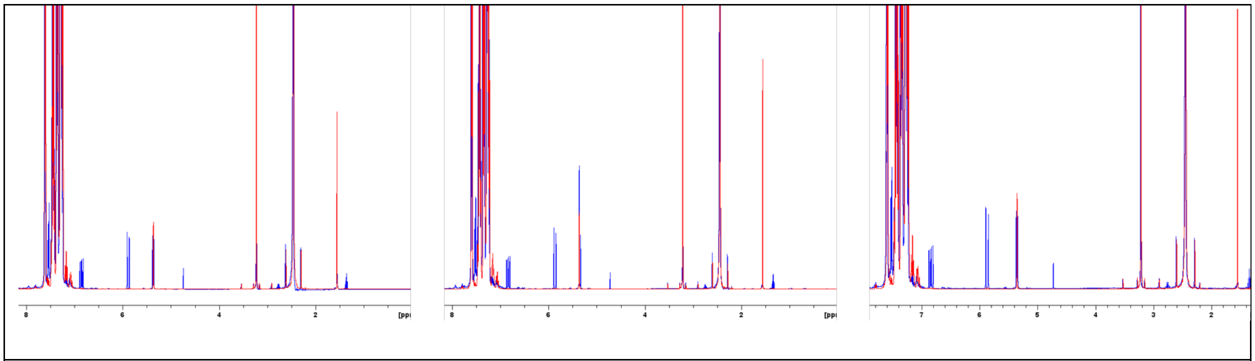

Figure S5. ^{1}H NMR spectra of hydrogenation of phenylacetylene by **1**. The red spectrum were measured before irradiation and the blue spectrum is measured after irradiation.

Quenching of PS by Organic Substrates

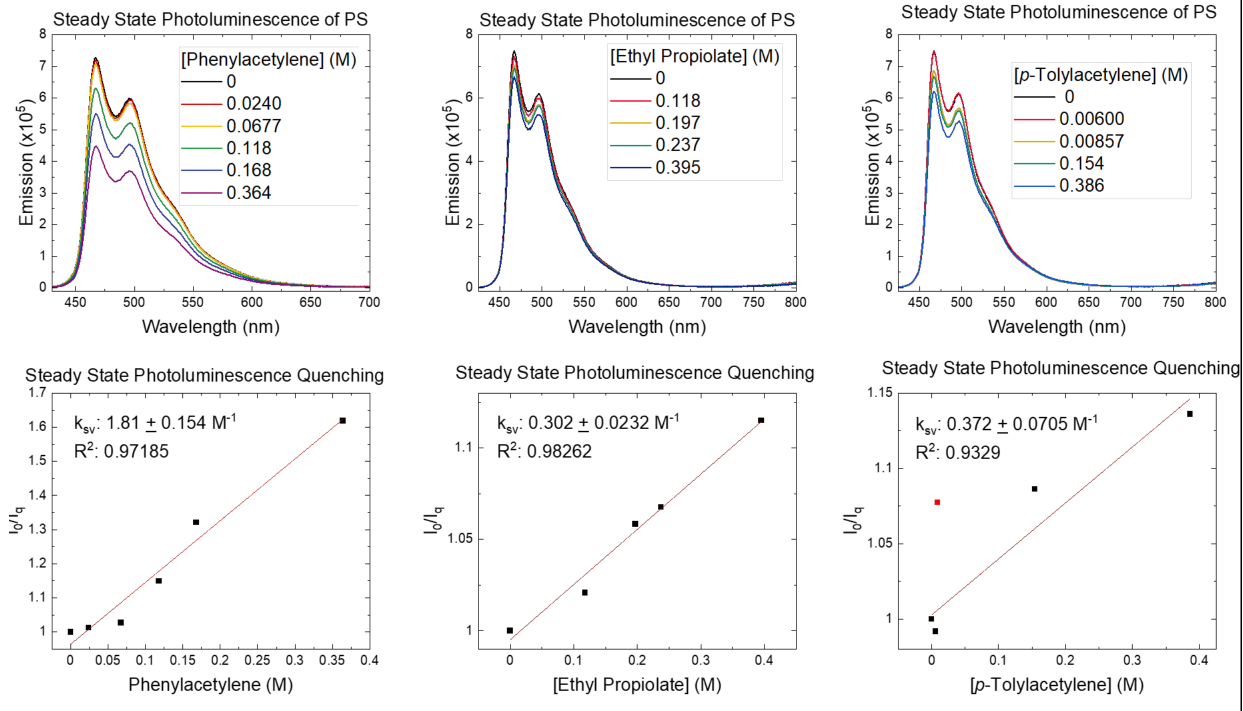

Figure S6. Static photoluminescence measuring Stern-Volmer quenching of **PS** by phenylacetylene, ethylpropiolate, or *p*-tolylacetylene (one point excluded from fit), exciting at 420 nm

Quenching Rate of PS by Organic Substrates

Substrate	k_q (M^{-1} s^{-1})
Phenylacetylene	(2.69 ± 0.229) x10^6
Ethylpropiolate	(4.49 ± 0.345) x10^5
p-Tolylacetylene	(5.54 ± 1.05) x10^5

^{1}H NMR Spectra

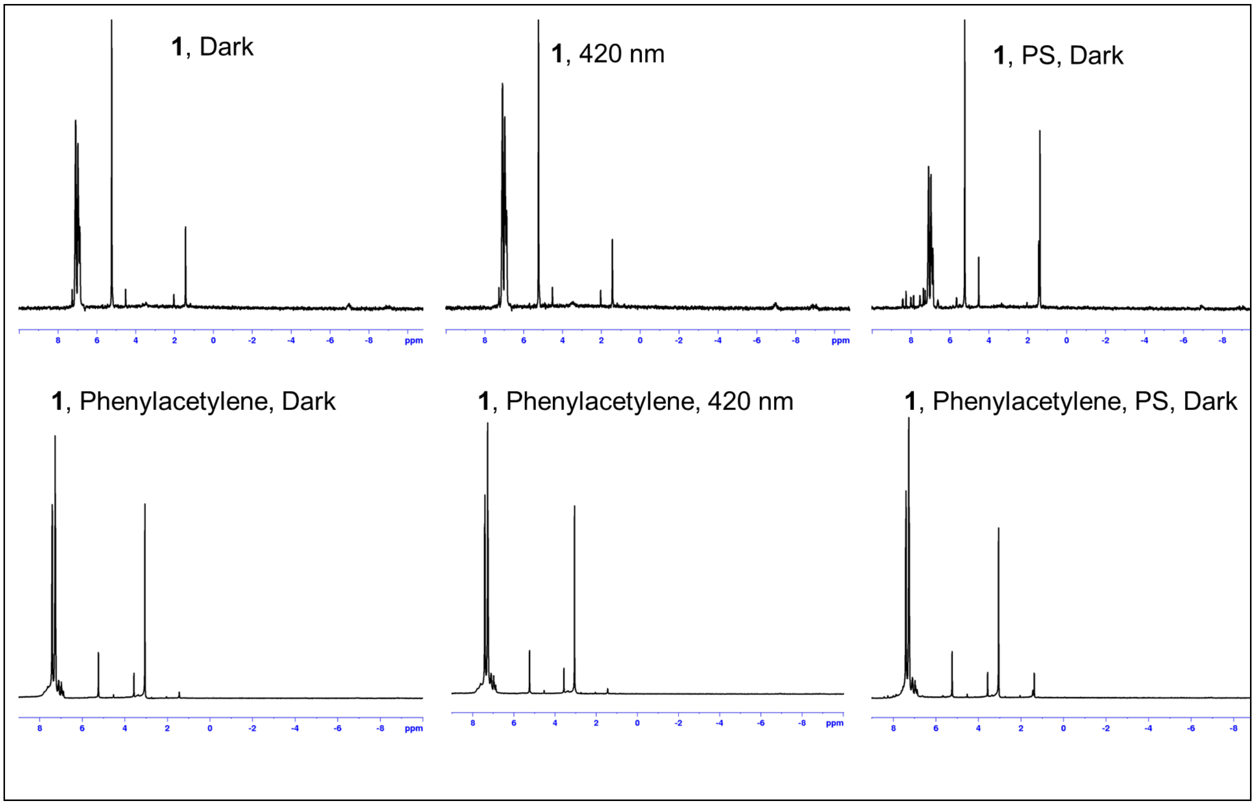

Figure S7. ^{1}H NMR spectrum of **1** and *p*-H_2 (top row, left to right): cycles in the dark, with 420 nm light, and with **PS** in the dark; and of **1**, *p*-H_2, and phenylacetylene (bottom row, left to right): in the dark, with 420 nm light, and with **PS** in the dark

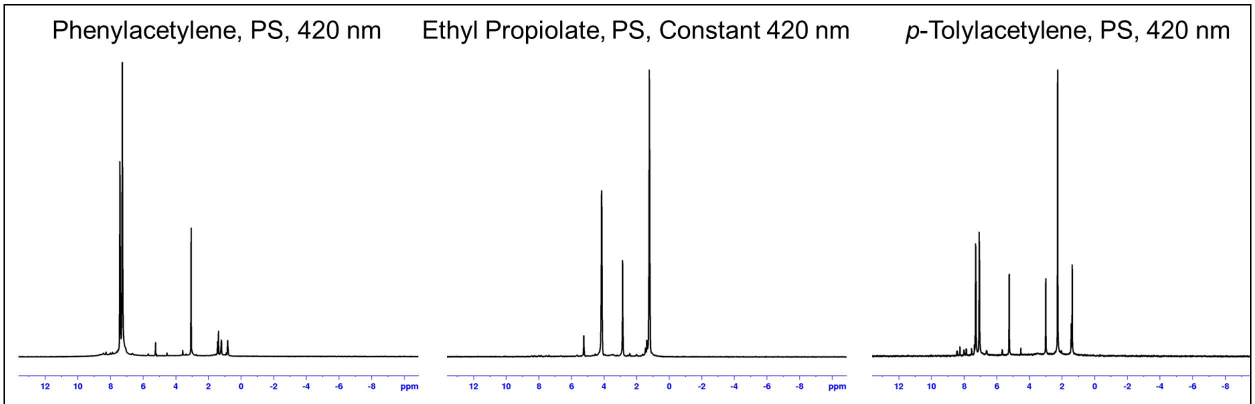

Figure S8. ^{1}H NMR spectrum of **PS** and 420 nm light with (left to right) phenylacetylene, ethylpropiolate, and *p*-tolylacetylene

Nuclear Spin Simulations. The spin evolution was simulated using the Spinach simulation library version 2.6.5625 in MATLAB (R2021a).[22,23] To match the experimental spectra, we varied the initial spin density matrix and simulated the resulting spectra after applying a 45° degree pulse. The chemical shifts and J-couplings used in these simulations for styrene are as follows: H_D (i=6.65 ppm), H_C (i=2, 5.68 ppm), H_B (i=3, 5.17 ppm) and H_D —H_C (-17.55 Hz), H_C —H_B (1.32 Hz), H_D —H_B (10.64 Hz).[24] The chemical shifts and J-couplings used in the simulations for ethylacrylate were the following: H_A (6.369 ppm), H_B (5.811 ppm), H_C (6.130 ppm), and H_A —H_B (2.75 Hz), H_A —H_C (9.69 Hz), and H_B —H_C (18.88 Hz), Figure S15. The chemical shifts and J-couplings used in

the simulations for 4-methylstyrene were the following: H_A (6.61 ppm), H_B (5.61 ppm), H_C (5.089 ppm), and H_A —H_B (17.567 Hz), H_A —H_C (10.943 Hz), and H_B —H_C (1.114 Hz), Figure S16.

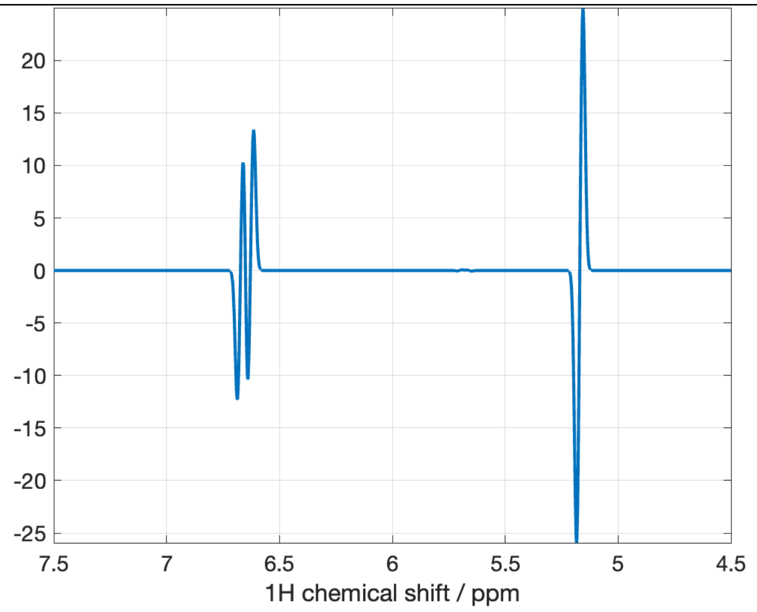

Figure S9. Pure cis addition, H_D and H_C for styrene

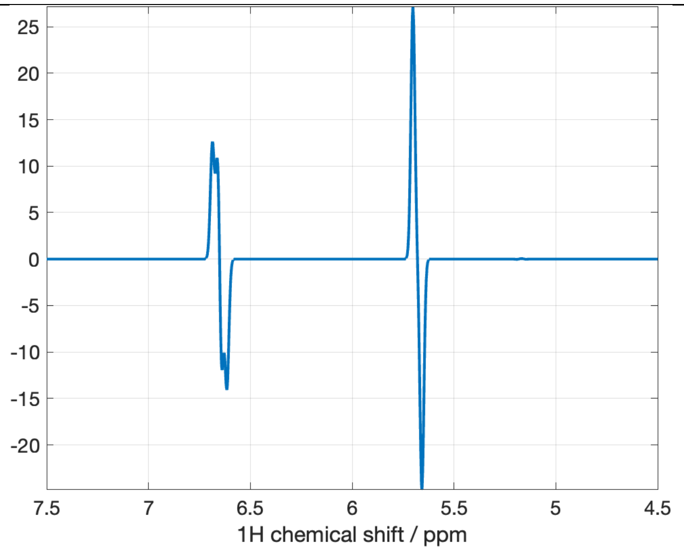

Figure S10. Pure trans addition: H_D and H_B for styrene

To demonstrate the lack of polarization transfer from singlet state on any spin pair we set up pure cis addition for styrene molecule, at t = 0 sec. **Figure S11** shows that there is no time evolution for spin system over 1 sec. In other words, polarization from singlet state on H_D and H_B protons does not transfer to other spin, H_C. Analogously, polarization from singlet state on H_D and H_C protons does not transfer to other spin, H_B.

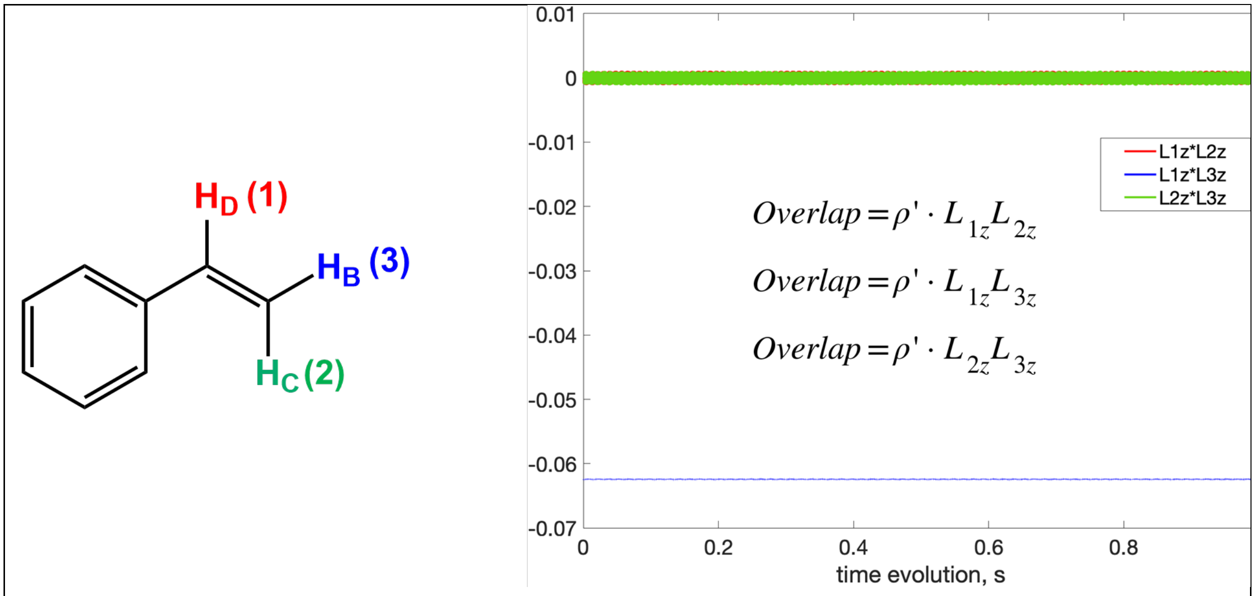

Figure S11. SPINACH calculations indicating lack of polarization transfer from singlet on any two specified protons after a 45° pulse. Here using styrene.

^{1}H NMR Spectra Styrene Exchange

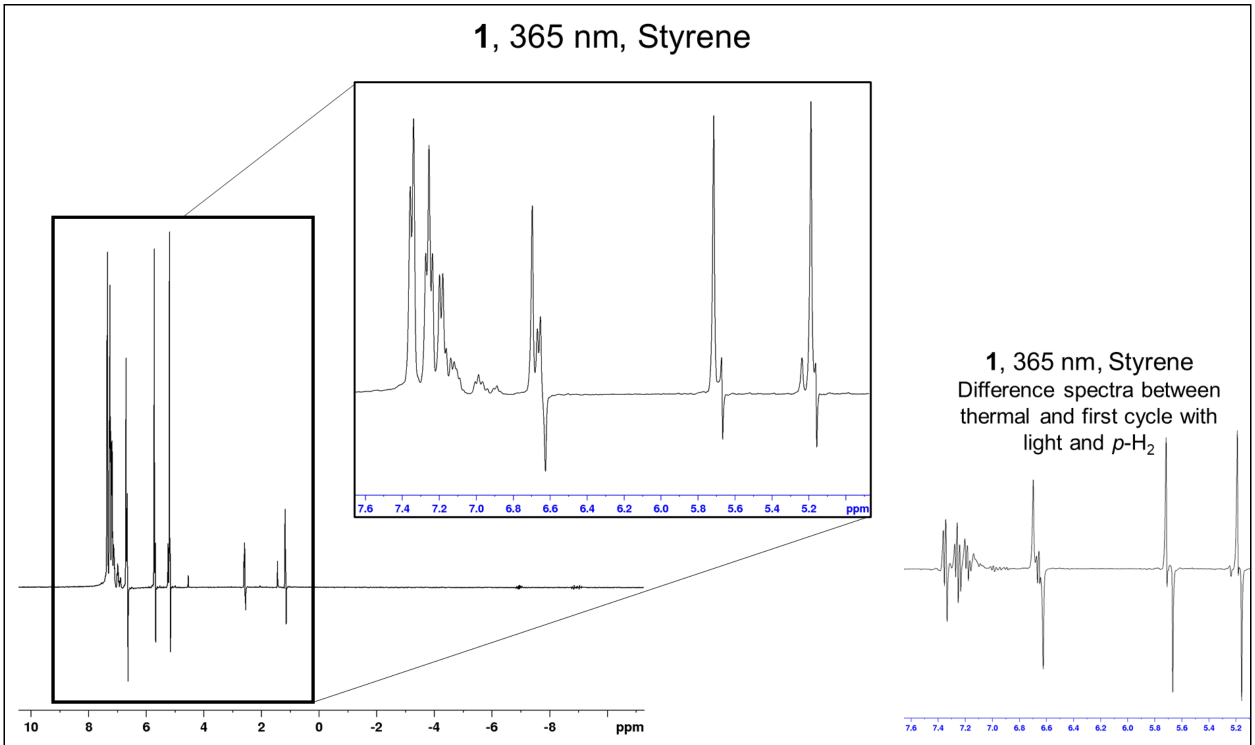

Figure S12. Styrene exchange with **1**, styrene, *p*-H_2, and 365 nm light

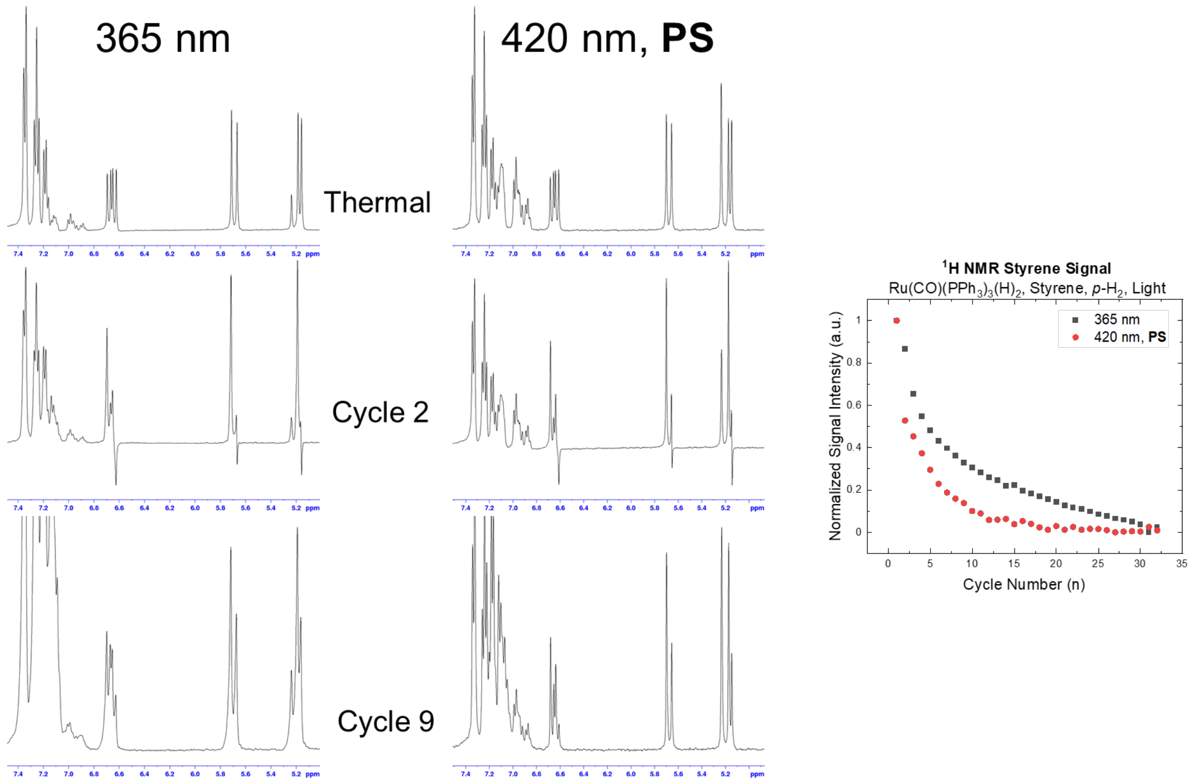

Figure S13. Styrene exchange with **1** and *p*-H_2 with 365 nm excitation and with **PS** and 420 nm light.

T_1 of Styrene

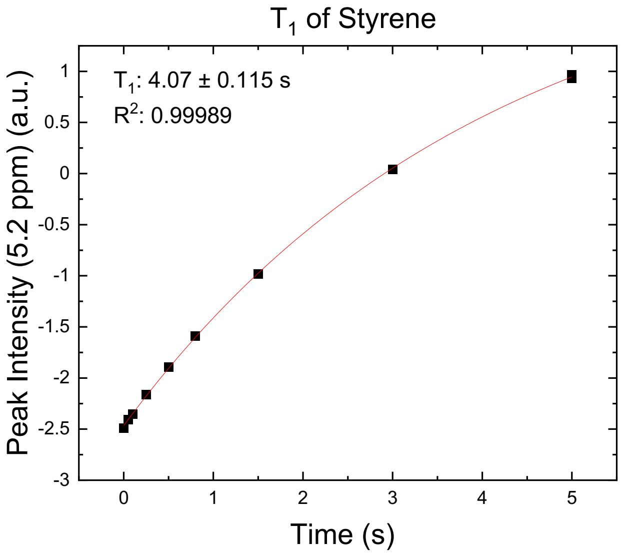

Figure S14. T_1 of Styrene in $CDCl_3$

^{1}H NMR Spectra with Selected Organic Substrates

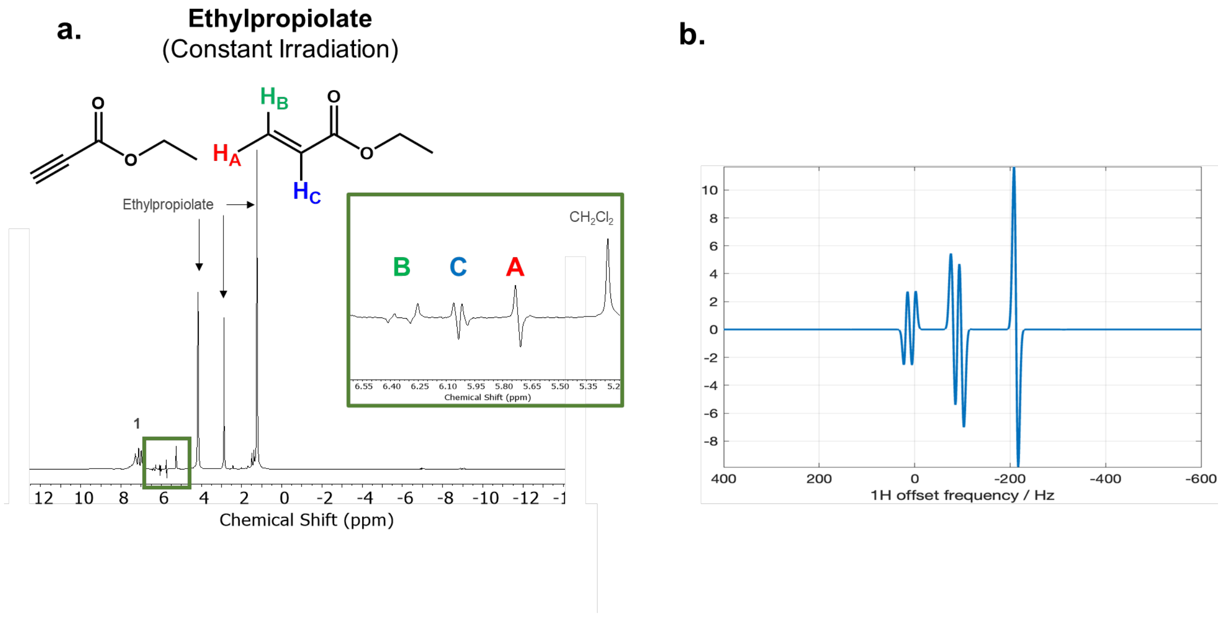

Figure S15. a. ^{1}H NMR spectrum of **1**, **PS,** 420 nm irradiation, p-H_2, and ethylpropiolate, and b. simulated spectra of ethylacrylate with 50% cis addition and 50% geminal addition

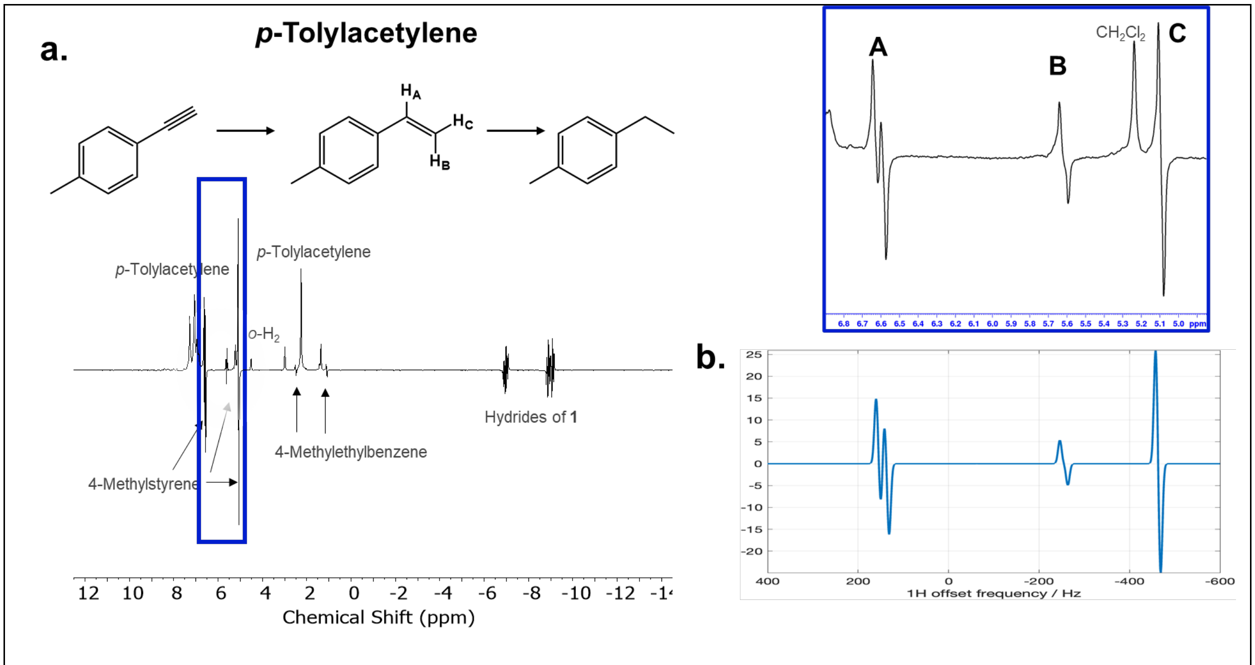

Figure S16. a. ^{1}H NMR spectrum of **1**, **PS,** 420 nm irradiation, *p*-H_2, and *p*-tolylacetylene. And b. simulated spectra of 4methylstyrene: 20% trans addition and 100% cis addition

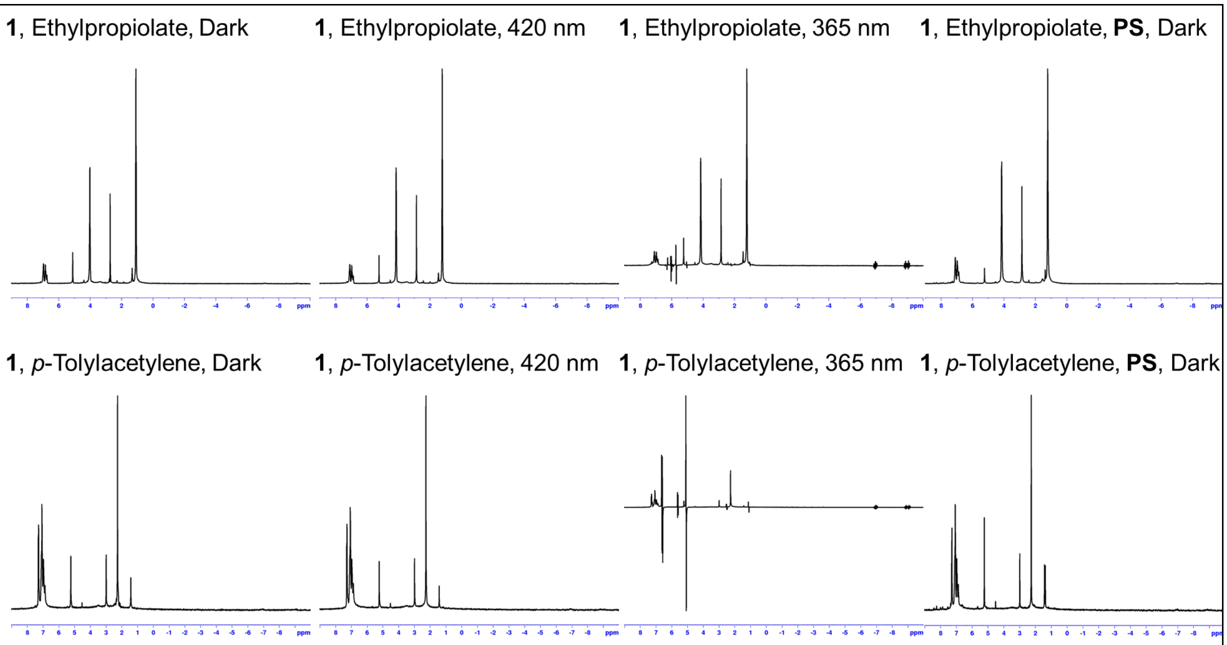

Figure S17. ^{1}H NMR spectrum of **1,** *p*-H_2, and ethylpropiolate (top row) or *p*-tolylacetlene (bottom row): (left to right) cycles in the dark, with 420 nm light, with 365 nm light, and with **PS** in the dark

Hyperpolarization at 0 °C

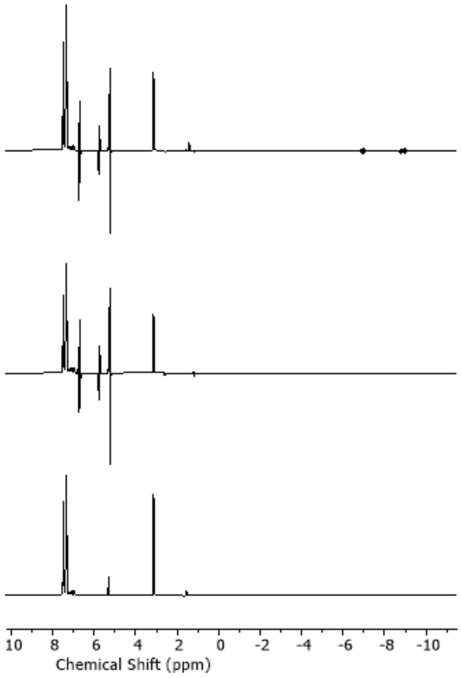

Figure S18. 0 °C hyperpolarization with **1**, phenylacetylene, top to bottom, **PS**, 420 nm light; 365 nm light; thermal spectrum.

REFERENCES

(1) Ahmad, N.; Levison, J. J.; Robinson, S. D.; Uttley, M. F.; Wonchoba, E. R.; Parshall, G. W. Complexes of Ruthenium, Osmium, Rhodium, and Iridium Containing Hydride Carbonyl, or Nitrosyl Ligands. In *Inorganic Syntheses*; John Wiley & Sons, Ltd, 1974; pp 45–64. https://doi.org/10.1002/9780470132463.ch13.

(2) TomHon, P.; Abdulmojeed, M.; Adelabu, I.; Nantogma, S.; Kabir, M. S. H.; Lehmkuhl, S.; Chekmenev, E. Y.; Theis, T. Temperature Cycling Enables Efficient ^{13}C SABRE-SHEATH Hyperpolarization and Imaging of [1-^{13}C]-Pyruvate. *J. Am. Chem. Soc.* **2022**, *144* (1), 282–287. https://doi.org/10.1021/jacs.1c09581.

(3) Hövener, J.-B.; Bär, S.; Leupold, J.; Jenne, K.; Leibfritz, D.; Hennig, J.; Duckett, S. B.; von Elverfeldt, D. A Continuous-Flow, High-Throughput, High-Pressure Parahydrogen Converter for Hyperpolarization in a Clinical Setting. *NMR Biomed.* **2013**, *26* (2), 124–131. https://doi.org/10.1002/nbm.2827.

(4) M. J. Frisch G. W. Trucks and H. B. Schlegel and G. E. Scuseria and M. A. Robb and J. R. Cheeseman and G. Scalmani and V. Barone and G. A. Petersson and H. Nakatsuji and X. Li and M. Caricato and A. V. Marenich and J. Bloino and B. G. Janesko and R. Gomperts and B. Mennucci and H. P. Hratchian and J. V. Ortiz and A. F. Izmaylov and J. L. Sonnenberg and D. Williams-Young and F. Ding and F. Lipparini and F. Egidi and J. Goings and B. Peng and A. Petrone and T. Henderson and D. Ranasinghe and V. G. Zakrzewski and J. Gao and N. Rega and G. Zheng and W. Liang and M. Hada and M. Ehara and K. Toyota and R. Fukuda and J. Hasegawa and M. Ishida and T. Nakajima and Y. Honda and O. Kitao and H. Nakai and T. Vreven and K. Throssell and Montgomery, {Jr.}, J. A. and J. E. Peralta and F. Ogliaro and M. J. Bearpark and J. J. Heyd and E. N. Brothers and K. N. Kudin and V. N. Staroverov and T. A. Keith and R. Kobayashi and J. Normand and K. Raghavachari and A. P. Rendell and J. C. Burant and S. S. Iyengar and J. Tomasi and M. Cossi and J. M. Millam and M. Klene and C. Adamo and R. Cammi and J. W. Ochterski and R. L. Martin and K. Morokuma and O. Farkas and J. B. Foresman and D. J. Fox. Gaussian 16.

(5) Becke, A. D. Density-functional Thermochemistry. III. The Role of Exact Exchange. *J. Chem. Phys.* **1993**, *98* (7), 5648–5652. https://doi.org/10.1063/1.464913.

(6) Hay, P. J.; Wadt, W. R. Ab Initio Effective Core Potentials for Molecular Calculations. Potentials for the Transition Metal Atoms Sc to Hg. *J. Chem. Phys.* **1985**, *82* (1), 270–283. https://doi.org/10.1063/1.448799.

(7) Wadt, W. R.; Hay, P. J. Ab Initio Effective Core Potentials for Molecular Calculations. Potentials for Main Group Elements Na to Bi. *J. Chem. Phys.* **1985**, *82* (1), 284–298. https://doi.org/10.1063/1.448800.

(8) T. H. Dunning Jr.; P. J. Hay. Modern Theoretical Chemistry. In *Modern Theoretical Chemistry*; Plenum, New York, 1977; Vol. 3, pp 1–28.

(9) *Ab initio effective core potentials for molecular calculations. Potentials for K to Au including the outermost core orbitals: The Journal of Chemical Physics: Vol 82, No 1*. https://aip.scitation.org/doi/10.1063/1.448975 (accessed 2022-06-20).

(10) *Self—Consistent Molecular Orbital Methods. XII. Further Extensions of Gaussian—Type Basis Sets for Use in Molecular Orbital Studies of Organic Molecules: The Journal of Chemical Physics: Vol 56, No 5*. https://aip.scitation.org/doi/10.1063/1.1677527 (accessed 2022-06-20).

(11) Rassolov, V. A.; Ratner, M. A.; Pople, J. A.; Redfern, P. C.; Curtiss, L. A. 6-31G* Basis Set for Third-Row Atoms. *J. Comput. Chem.* **2001**, *22* (9), 976–984. https://doi.org/10.1002/jcc.1058.

(12) Blaudeau, J.-P.; McGrath, M. P.; Curtiss, L. A.; Radom, L. Extension of Gaussian-2 (G2) Theory to Molecules Containing Third-Row Atoms K and Ca. *J. Chem. Phys.* **1997**, *107* (13), 5016–5021. https://doi.org/10.1063/1.474865.

(13) Binning Jr., R. C.; Curtiss, L. A. Compact Contracted Basis Sets for Third-Row Atoms: Ga–Kr. *J. Comput. Chem.* **1990**, *11* (10), 1206–1216. https://doi.org/10.1002/jcc.540111013.

(14) Ditchfield, R.; Hehre, W. J.; Pople, J. A. Self-Consistent Molecular-Orbital Methods. IX. An Extended Gaussian-Type Basis for Molecular-Orbital Studies of Organic Molecules. *J. Chem. Phys.* **1971**, *54* (2), 724–728. https://doi.org/10.1063/1.1674902.

(15) Francl, M. M.; Pietro, W. J.; Hehre, W. J.; Binkley, J. S.; Gordon, M. S.; DeFrees, D. J.; Pople, J. A. Self-consistent Molecular Orbital Methods. XXIII. A Polarization-type Basis Set for Second-row Elements. *J. Chem. Phys.* **1982**, *77* (7), 3654–3665. https://doi.org/10.1063/1.444267.

(16) Gordon, M. S. The Isomers of Silacyclopropane. *Chem. Phys. Lett.* **1980**, *76* (1), 163–168. https://doi.org/10.1016/0009-2614(80)80628-2.
(17) Hariharan, P. C.; Pople, J. A. Accuracy of AH n Equilibrium Geometries by Single Determinant Molecular Orbital Theory. *Mol. Phys.* **1974**, *27* (1), 209–214. https://doi.org/10.1080/00268977400100171.
(18) Hariharan, P. C.; Pople, J. A. The Influence of Polarization Functions on Molecular Orbital Hydrogenation Energies. *Theor. Chim. Acta* **1973**, *28* (3), 213–222. https://doi.org/10.1007/BF00533485.
(19) Arias-Rotondo, D. M.; McCusker, J. K. The Photophysics of Photoredox Catalysis: A Roadmap for Catalyst Design. *Chem. Soc. Rev.* **2016**, *45* (21), 5803–5820. https://doi.org/10.1039/C6CS00526H.
(20) TomHon, P.; Akeroyd, E.; Lehmkuhl, S.; Chekmenev, E. Y.; Theis, T. Automated Pneumatic Shuttle for Magnetic Field Cycling and Parahydrogen Hyperpolarized Multidimensional NMR. *J. Magn. Reson.* **2020**, *312*, 106700. https://doi.org/10.1016/j.jmr.2020.106700.
(21) Tse, S. K. S.; Xue, P.; Lin, Z.; Jia, G. Hydrogen/Deuterium Exchange Reactions of Olefins with Deuterium Oxide Mediated by the Carbonylchlorohydrido-Tris(Triphenylphosphine)Ruthenium(II) Complex. *Adv. Synth. Catal.* **2010**, *352* (9), 1512–1522. https://doi.org/10.1002/adsc.201000037.
(22) Hogben, H. J.; Krzystyniak, M.; Charnock, G. T. P.; Hore, P. J.; Kuprov, I. Spinach – A Software Library for Simulation of Spin Dynamics in Large Spin Systems. *J. Magn. Reson.* **2011**, *208* (2), 179–194. https://doi.org/10.1016/j.jmr.2010.11.008.
(23) SpinDynamics.Org – Spin Dynamics Group.
(24) Nies, H.; Bauer, H.; Roth, K.; Rewicki, D. The Complete 1H Spectral Analysis of Styrene. *J. Magn. Reson. 1969* **1980**, *39* (3), 521–524. https://doi.org/10.1016/0022-2364(80)90038-4.

.
